# Comparative evaluation of reference-free transcriptomic deconvolution highlights the importance of biological validation in astrocytes across Alzheimer’s disease

**DOI:** 10.3389/fbinf.2026.1858866

**Published:** 2026-07-13

**Authors:** Laura Rodríguez-Millan, Andrea Angarita-Rodríguez, Viviana Vargas-López, Andrés Pinzón, Estefania Tarifeño-Saldivia, Janneth González

**Affiliations:** 1 Departamento de Nutrición y Bioquímica, Facultad de Ciencias, Pontificia Universidad Javeriana, Bogotá, Colombia; 2 Laboratorio de Bioinformática y Biología de Sistemas, Universidad Nacional de Colombia Bogotá, Bogotá, Colombia; 3 Department of Biochemistry and Molecular Biology, Faculty of Biological Sciences, University of Concepción, Concepción, Chile

**Keywords:** Alzheimer’s disease, astrocytes, bulk RNA-seq, CDSeq, cognitive impairment, DECODER, transcriptomic deconvolution

## Abstract

**Introduction:**

Astrocytes are central regulators of neuronal energy metabolism and redox homeostasis—processes that become progressively disrupted across the Alzheimer’s disease (AD) continuum. However, bulk transcriptomic data obscure cell-type–specific signals, and existing reference-free deconvolution methods often prioritize either statistical robustness or quantitative accuracy without fully integrating both dimensions.

**Methods:**

Here, we present a comparative framework evaluating two complementary unsupervised approaches, CDSeq and DECODER, to reconstruct astrocyte-associated transcriptomic profiles from human hippocampal samples spanning control, mild cognitive impairment (incipient and moderate), and AD (severe) stages (GSE28146; n = 30). The inferred profiles were functionally contextualized through integration into a genome-scale metabolic model of human astrocytes, enabling the assessment of system-level metabolic alterations associated with disease progression.

**Results:**

Our results reveal consistent dysregulation of key astrocytic pathways, including impairment of the astrocyte–neuron lactate shuttle, disruption of glutamine metabolism, and reduced glutathione-mediated an oxidant capacity. Methodological benchmarking showed distinct yet complementary performance profiles: DECODER achieved higher accuracy in reconstructing global expression magnitudes, whereas CDSeq exhibited greater stability and preservation of gene–gene relationships. Crucially, external validation using independent single-nucleus RNA-seq astrocyte data demonstrated that CDSeq-derived profiles achieve moderate but robust concordance with reference signatures (r ≈ 0.43–0.44), substantially exceeding DECODER-derived concordance (r ≈ 0.19–0.23), with higher concordance with astrocyte-associated signatures, alongside preservation of canonical astrocyte markers and enrichment of astrocyte-specific pathways, indicating superior biological coherence.

**Discussion:**

Together, these findings demonstrate that technical accuracy does not necessarily translate into biological validity and highlight CDSeq as the method that more reliably captures astrocyte-specific transcriptional programs in this context. While DECODER remains valuable for detecting absolute expression changes, CDSeq provides a more consistent recovery of astrocyte-associated transcriptional patterns. More broadly, our results support the incorporation of biological validation alongside statistical benchmarking when selecting deconvolution methods for downstream systems biology and metabolic modeling applications. This framework establishes a reproducible strategy for evaluating deconvolution methods and their functional consequences, advancing the interpretation of bulk transcriptomic data in neurodegenerative disease.

## 1 Introduction

Alzheimer’s disease (AD) is characterized by a complex spatial and temporal cellular remodeling that is often obscured in bulk transcriptomic studies. High-throughput studies enable systematic characterization of cell-type–specific expression patterns, regulatory networks, and mechanisms involved in neurodegeneration (Alonso-Moreda et al., 2023; Avila Cobos et al., 2020). These methods have supported the discovery of molecular signatures and biomarkers relevant to diagnosis and therapy in Alzheimer’s disease (AD) (Chen S. et al., 2022).

Functional interpretation of bulk transcriptomic data remains challenging. The aggregate nature of bulk RNA-seq confounds cell-specific regulatory signals, masking the metabolic shifts that drive neurodegeneration. Bulk profiles integrate signals from multiple cell types and therefore obscure cell-specific alterations that drive disease progression (Guebel, 2023). This limitation is aggravated by the complex cellular composition of the neurovascular unit, which includes neurons, astrocytes, microglia, endothelial cells, and pericytes. Cell–cell interactions regulate metabolic support, synaptic function, and inflammatory tone, thereby influencing the initiation and progression of mild cognitive impairment (MCI) and AD (Guebel, 2023).

Astrocytes are central regulators of cerebral energy and redox homeostasis. They modulate synaptic transmission via glutamate uptake and recycling and provide metabolic substrates that sustain neuronal activity (Escartin et al., 2019; Magistretti and Allaman, 2015). Alterations in these astroglial functions contribute to synaptic deterioration and cognitive decline during MCI and AD.

To address cellular mixing, computational transcriptomic deconvolution tools (TDTs) estimate cell-type proportions or infer cell-type–specific expression from bulk data. TDTs include supervised methods (require reference signatures), semi-supervised approaches (combine references and data-driven features), and unsupervised or *de novo* methods (no reference required) (Nguyen et al., 2024). However, supervised approaches rely on external reference signatures that are often derived from healthy tissues or animal models, which may not capture disease-specific transcriptional states. Using healthy references to deconvolve diseased brains can therefore introduce systematic biases. Although the growing availability of Alzheimer’s disease single-cell and single-nucleus RNA-seq datasets has substantially improved the feasibility of reference-based deconvolution, important challenges remain regarding the transferability of reference signatures across cohorts, brain regions, disease stages, sequencing platforms, and cell-state annotation strategies. In particular, reactive astrocyte states exhibit considerable transcriptional heterogeneity across studies, potentially limiting the generalizability of fixed reference matrices in independent bulk transcriptomic datasets. Examples of unsupervised tools are DECODER and CDSeq, which have shown promising results in complex tissues where complete references are scarce (Kang et al., 2019). Importantly, reference-free deconvolution approaches infer latent transcriptomic compartments that may reflect cell-type–enriched programs, reactive cellular states, or shared stress-associated processes rather than perfectly discrete cellular identities. Accordingly, these limitations support the use of reference-free approaches for recovering biologically coherent astrocyte-associated programs under heterogeneous neurodegenerative conditions. A systematic survey of available transcriptomic datasets and reference resources used in this study is provided in Supplementary Table S1.

Most deconvolution studies focus on estimating cell proportions and rarely translate inferred profiles into functional consequences. Integrating inferred profiles with genome-scale metabolic models bridges this gap. This strategy maps expression changes to reaction constraints and enables the identification of altered pathways and candidate functional drivers of energetic failure and oxidative stress (Angarita-Rodríguez et al., 2024).

Here, we pursue three objectives: (i) systematically benchmarking and prioritizing transcriptomic deconvolution tools suitable for heterogeneous human hippocampal bulk data; (ii) implement and benchmark two top unsupervised tools (CDSeq and DECODER) against single-cell–derived astrocyte references and simulated pseudobulks from GSE28146 (Blalock et al., 2011); and (iii) integrate the inferred astrocyte-specific profiles into an updated genome-scale astrocyte model to assess functional consequences of disease-related expression changes.

## 2 Materials and methods

### 2.1 Astrocyte-specific transcriptomic profiling and data preprocessing

Astrocyte-specific profiling was performed using human hippocampal transcriptomic data (CA1–CA3; mean age 86.3 ± 1.4 years) derived from the GSE28146 dataset (GSE28146; Blalock et al., 2011), which comprises 30 post-mortem samples from individuals spanning different clinical stages, including control (n = 8), incipient (n = 7), moderate (n = 8), and severe AD (n = 7).

Clinical groups in GSE28146 were defined according to the original study labels. To provide clinical context, Mini-Mental State Examination (MMSE) scores were used as descriptive indicators of cognitive impairment severity rather than strict classification criteria. The approximate MMSE ranges associated with each stage are as follows: healthy controls (MMSE ≥25), early MCI (MMSE 20–24), advanced MCI (MMSE 14–19), and Alzheimer’s disease (MMSE ≤14).

Microarray preprocessing was performed using the affy and limma Bioconductor packages, including Robust Multi-array Average (RMA) background correction and quantile normalization across arrays (Bumgarner, 2014; Whitney et al., 1999). As RMA produces log-transformed expression values, these were exponentiated to obtain non-negative intensity values, which were used as input for CDSeq. While this transformation enables compatibility with the deconvolution framework, these transformed intensities do not reproduce the statistical properties of RNA-seq count distributions and may introduce distributional bias during probabilistic deconvolution, which should be considered when interpreting the results (Kang et al., 2021).

This scaling strategy harmonized the dynamic range of microarray intensities with deconvolution algorithm requirements while preserving biologically informative relative expression patterns (Kang et al., 2021). Although the resulting values do not correspond to true count data, they retain transcriptional directionality, enabling the propagation of metabolic constraints through Gene–Protein–Reaction (GPR) associations within the genome-scale model (Filippo et al., 2021).

### 2.2 Identification of transcriptomic deconvolution tools

To identify optimal TDTs for the transcriptomic decomposition of hippocampal tissue, we implemented a systematic multi-criteria benchmarking protocol spanning 2010–2024. Searches were performed in PubMed, Web of Science, and Scopus using combinations of keywords including “transcriptomic deconvolution,” “bulk RNA-seq,” “cell type composition,” and “brain.”

35 TDTs were identified and compiled into a database annotated according to their technical characteristics (Supplementary Table S2). Based on this database, a weighted feature matrix was constructed (Supplementary Table S3). Each tool was scored according to its applicability to hippocampal tissue, considering tolerance to cellular heterogeneity, code availability, algorithmic class, dependence on prior information, sensitivity, scalability, documentation quality, and the underlying mathematical framework (Supplementary Table S2). The selection of datasets and transcriptomic resources considered during this benchmarking process is summarized in Supplementary Table S1.

Tools and features were encoded as a bipartite network (features → tools). This representation enabled the identification of methods that maximize unsupervised signal disentanglement under high hippocampal heterogeneity, while prioritizing stability for downstream Systems Biology applications and constraint propagation in genome-scale metabolic models (GEMs).

Topological relevance was quantified through centrality indices, including weighted degree, degree, and betweenness centrality. Analyses were implemented in Python v3.14.0 using the NetworkX library (Supplementary Table S4). The resulting network was visualized as a bibliometric map using VOSviewer v1.6.20 (van Eck and Waltman, 2010). A minimum total link strength threshold of five was applied, yielding 73 nodes.

This integrative framework identified DECODER and CDSeq as the most robust TDTs for reference-free deconvolution of hippocampal transcriptomes (Supplementary Figure S1).

### 2.3 Implementation of TDTs

Astrocytic transcriptomic profiles were reconstructed across the clinical continuum of Alzheimer’s disease, spanning control, incipient, moderate, and severe stages, using hippocampal bulk transcriptomic intensities. Expression matrices were transformed into pseudocounts and deconvolved using transcriptomic deconvolution tools with empirically optimized convergence criteria to maximize reconstruction fidelity and ensure model stability.

Because CDSeq was originally designed for count-based RNA-seq data, application to microarray-derived expression profiles required transformation of RMA-normalized non-negative intensities into pseudocount-like matrices to ensure compatibility with the algorithmic framework. Although this transformation preserves relative transcriptomic structure across samples, it does not fully reproduce the statistical properties of true sequencing count distributions and may therefore influence parameter estimation and convergence behavior. Parameter optimization was therefore performed independently for each deconvolution framework according to its underlying mathematical structure. Parameter optimization was therefore performed independently for each deconvolution framework according to its underlying mathematical structure.

We acknowledge that CDSeq and DECODER were implemented under different modeling strategies (condition-specific vs. global decomposition), which may introduce methodological asymmetry. While this design reflects the intrinsic algorithmic structure of each method and was chosen to optimize convergence stability and biological interpretability within each framework, it may also influence direct quantitative comparability between methods. Therefore, this limitation should be considered when interpreting benchmarking results.

For DECODER, the number of latent factors (K) was selected using cophenetic correlation stability combined with biological interpretability of the inferred compartments, whereas the number of latent transcriptomic components (T) in CDSeq was optimized independently across clinical conditions to improve convergence stability and preservation of biologically coherent astrocyte-associated transcriptional structure under varying levels of transcriptomic heterogeneity. Accordingly, CDSeq-derived outputs were interpreted as biologically informative latent transcriptomic compartments rather than exact probabilistic reconstructions of cell-type composition.

The inferred compartments were functionally characterized using enrichment analyses performed with the Metascape platform (Zhou et al., 2019). This analysis identified molecular functions associated with hippocampal astrocytes across clinical stages.

Because both CDSeq and DECODER operate in a fully unsupervised setting, inferred latent components were not assumed *a priori* to correspond to discrete astrocyte transcriptomes. Instead, astrocyte-associated compartments were identified through a multi-level annotation strategy integrating canonical astrocyte marker expression, functional enrichment analyses, concordance with external astrocyte single-nucleus RNA-seq references, and preservation of disease-stage transcriptional structure. However, this annotation framework may introduce a potential selection bias toward previously characterized astrocytic signatures, which should be considered when interpreting the specificity of the identified compartments.

Astrocyte-associated signals were prioritized based on the coordinated enrichment of canonical astrocyte markers, including well-established genes involved in neurotransmitter cycling, metabolic support, and redox homeostasis (Nelms et al., 2016; Escartin et al., 2019; Brugulat-Serrat et al., 2023) (Supplementary Table S5). These markers were integrated with pathway-level enrichment analyses and external reference datasets to strengthen biological interpretability and ensure consistency with known astrocytic functions. Importantly, this multi-layered validation framework allowed us to distinguish biologically meaningful signals from method-specific artifacts, reinforcing the robustness of the inferred astrocyte-specific transcriptional profiles across conditions.

Following component identification, a single astrocyte-associated compartment was selected per method and condition based on a composite scoring strategy integrating (i) astrocyte marker enrichment, (ii) correlation with astrocyte reference profiles, and (iii) biological pathway coherence.

To reduce model complexity and improve numerical stability, CDSeq was executed independently for each clinical cohort, allowing the model to capture condition-specific transcriptional patterns without imposing a shared structure across disease stages. This strategy enabled preservation of condition-specific latent transcriptional structure across disease progression, facilitating the identification of stage-dependent molecular signatures. However, this approach may favor condition-specific signal preservation at the expense of direct comparability with the globally decomposed DECODER framework, which operates under a unified reference structure across all samples.

### 2.4 DECODER

DECODER was applied in *de novo* mode (Peng et al., 2019) using MATLAB R2025a to identify transcriptomic compartments. The algorithm decomposes the expression matrix into two components: W (genes × factors), which represents gene loadings for each compartment, and H (factors × samples), which captures the relative contribution of each compartment across samples.

A range of factor numbers (K = 2–15) was evaluated. For each K value, 750 bootstrap replicates were performed to account for the limited sample size (n = 30). While increasing K allows for finer resolution of transcriptomic compartments, excessively high values may lead to over-fragmentation and reduced stability of the inferred components (Peng et al., 2019).

The selection of K was not based on a strict assumption of discrete clustering but rather guided by stability-based metrics such as the cophenetic correlation coefficient and inspection of consensus co-occurrence matrices, following Metagenes and molecular pattern discovery using matrix factorization (Janiesch and Heinrich, 2021). We acknowledge that such metrics were originally developed for clustering contexts and may not fully capture the continuous mixture nature of deconvolution models.

Therefore, K selection was complemented by biological interpretability and downstream validation analyses, in line with approaches used in dynamic deconvolution frameworks such as DECODER deconvolution method (Supplementary Figure S1).

DECODER integrates non-negative matrix factorization (NMF) with non-negative least squares (NNLS) refinement. Bootstrap submatrices were generated from the original expression matrix A′ (genes × samples), followed by repeated NMF decompositions for each K value (Peng et al., 2019). Marker genes were identified for each factor, and a co-occurrence matrix was constructed to derive a consensus factor seed. This seed was then used for final NMF–NNLS optimization.

This iterative strategy improves numerical convergence and reduces decomposition variability across runs. It enables the identification of consistent transcriptomic compartments in heterogeneous tissues such as the hippocampus (Peng et al., 2019; Repsilber et al., 2010).

Astrocyte-specific marker genes identified from these compartments were subsequently used as seed inputs for GEM analyses.

### 2.5 CDSeq

CDSeq (Cell-type Deconvolution using Sequencing data) was applied in a fully unsupervised manner using the CDSeqR package in R (Kang et al., 2021). The method was used to infer cell-type–specific gene expression profiles and sample-specific cell-type proportions from bulk transcriptomic data.

A range of cell types (T = 2–20) was evaluated using 1,000 Gibbs sampling iterations per run (Kang et al., 2021). This broader range, compared to the factor exploration performed in DECODER, was selected to account for the increased flexibility of CDSeq in modeling fine-grained transcriptomic components under a probabilistic framework.

CDSeq was executed independently for each clinical cohort, allowing the model to capture condition-specific transcriptional patterns without imposing a shared structure across disease stages. This strategy enabled preservation of condition-specific latent transcriptional structure across disease progression, facilitating the identification of stage-dependent molecular signatures. As a result, the inferred cell-type–specific profiles reflect not only common astrocytic features but also dynamic changes associated with disease severity, supporting their use for downstream comparative and mechanistic analyses.

CDSeq relies on a hierarchical Bayesian framework (Kang et al., 2021). Gene read counts are modeled as a multinomial mixture of latent cell-type components. Each component is defined by a gene expression profile (φ) and a sample-specific proportion vector (θ). Both latent matrices are inferred via Gibbs sampling under Dirichlet priors. This formulation captures biological variability while preserving relative expression structure across genes and samples. Model convergence was assessed by monitoring stabilization of log-likelihood trajectories across iterations.

Because CDSeq operates in a reference-free setting, label switching across inferred components represents a known limitation, particularly in complex tissues such as the brain. To mitigate label switching during MCMC sampling, inferred components were aligned *post hoc* using a maximum-weight matching strategy guided by the biological coherence of canonical astrocyte markers.

Overall, this MCMC-based probabilistic framework enables flexible inference of cell-type proportions and expression profiles in highly heterogeneous tissues. However, performance depends on prior specification, which can influence convergence behavior and bias parameter estimates (Kang et al., 2019; Li et al., 2013; Li and Xie, 2013; Wang et al., 2018). In contrast, CDSeq preserves relative expression structure through Dirichlet–MCMC priors, favoring system-level stability, whereas DECODER employs deterministic NMF–NNLS projections to prioritize fidelity in absolute expression magnitude.

### 2.6 Statistical analysis

Following deconvolution, the accuracy and concordance of the estimated expression profiles were evaluated using Pearson correlation, Spearman correlation, and root mean square error (RMSE) (Stumpf et al., 2011). Bulk transcriptomic data were obtained from GSE28146 (Blalock et al., 2011), comprising hippocampal CA1 samples across control, incipient MCI, advanced MCI, and AD. As reference, transcriptomic profiles were derived from the Human Brain Cell Atlas v1.0 (healthy human hippocampus), available through the Allen Institute for Brain Science/Human Cell Atlas consortium (Human Brain Cell Atlas v1.0). This dataset includes aggregated gene expression profiles for major human brain cell types, including astrocytes, pyramidal neurons, oligodendrocytes, microglia, and endothelial cells.

Four major cell types were selected for validation: interneurons, pyramidal neurons, astrocytes, and microglia. The gene expression matrix was filtered to exclude mitochondrial and ribosomal transcripts, as well as lowly expressed genes detected in fewer than 5 cells.

A reference astrocyte pseudobulk was constructed by averaging gene expression across astrocyte cells. Astrocytic identity was verified using a curated set of marker genes (S100B, GFAP, SLC1A3, FGFR3, AQP4, GLUL, and ALDH1L1). The resulting reference matrix contained 16,176 genes.

Outputs from DECODER and CDSeq were processed to generate comparable pseudobulks for each clinical condition (control, incipient, moderate, and severe). For DECODER, the sample_w and gene_w matrices were normalized by rows and columns to unit sum (∑ = 1), and their matrix product yielded a simulated expression matrix that was subsequently averaged by clinical condition (Peng et al., 2019). CDSeq directly produced condition-specific expression matrices, which were already normalized expression profiles were normalized using total sum scaling to represent relative abundance and subsequently transformed using log_2_ (x + 1) to stabilize variance across clinical conditions (Kang et al., 2021).

### 2.7 Accuracy and concordance analysis

Reconstruction accuracy was quantified by benchmarking control-condition pseudobulks against single-cell–derived astrocyte reference profiles obtained from the Human Brain Cell Atlas v1.0 (healthy human hippocampus), available through the Human Cell Atlas Data Portal (Human Brain Cell Atlas v1.0). Concordance between DECODER and CDSeq was evaluated across clinical conditions to assess methodological consistency. These metrics collectively captured linear dependency, monotonic relationship consistency, and absolute deviation in expression magnitude.

Importantly, all validation analyses were anchored in the control condition prior to disease-stage comparisons. This design allowed us to evaluate whether inferred compartments correspond to expected astroglial transcriptional programs under baseline physiological conditions, thereby providing a reference framework for interpreting disease-associated deviations.

To further evaluate the cell-type specificity and biological coherence of the inferred transcriptomic compartments, deconvolved profiles generated by CDSeq and DECODER were assessed using canonical marker gene sets representative of major brain cell populations, including astrocytes, neurons, microglia, and oligodendrocytes. Marker enrichment scores were calculated as the mean normalized expression of curated cell-type-specific genes and subsequently standardized (z-score) across conditions to enable comparative visualization. CDSeq-derived profiles exhibited strong and consistent relative enrichment of astrocytic markers across all clinical stages, together with comparatively lower enrichment of neuronal and microglial signatures, suggesting improved segregation of astrocyte-associated transcriptional programs. In contrast, DECODER-derived profiles showed a more heterogeneous enrichment pattern, with detectable overlap among astrocytic, neuronal, and oligodendrocyte-associated markers, indicative of reduced compartmental resolution. Additionally, astrocyte marker dynamics across Alzheimer’s disease progression revealed that CDSeq preserved structured stage-dependent transcriptional variation, whereas DECODER displayed a comparatively uniform signal across conditions. Collectively, these analyses supported the use of CDSeq-derived compartments for downstream astrocyte-focused metabolic modeling.

### 2.8 Robustness analysis

Algorithmic robustness was evaluated following the benchmarking framework proposed by (Hu and Chikina, 2024), focusing on stability under controlled stochastic perturbations. A synthetic pseudobulk dataset of 50 samples was generated for the four selected hippocampal cell types. Biological variance was modeled through Dirichlet–multinomial sampling and intra–cell-type stochastic subsampling to recapitulate the compositional heterogeneity of hippocampal tissue, while technical variability was simulated through differences in sequencing depth, read counts, and dropout rates. The simulation framework was designed to reproduce biologically realistic transcriptomic variability under controlled perturbation conditions. Dataset preprocessing and normalization procedures were applied consistently across all samples to minimize technical variability and ensure comparability across simulated conditions.

Simulated pseudobulks were analyzed using DECODER and CDSeq with the same parameters described previously. Robustness was quantified using Pearson and Spearman correlations, as well as RMSE. This analysis assessed algorithm stability under combined biological and technical variability, supporting their applicability to hippocampal transcriptomic data.

### 2.9 Integration of expression profiles into the astrocyte metabolic model

To reconstruct context-specific metabolic flux distributions from the transcriptomic profiles derived via CDSeq and DECODER, expression data were integrated into a curated genome-scale metabolic model (GEM) of the human astrocyte (Angarita-Rodríguez et al., 2024). The GEM was previously validated for energy metabolism and antioxidant capacity, supporting its suitability for simulating hippocampal astrocyte homeostasis under neurodegenerative constraints.

For each deconvolution tool, four context-specific metabolic models were generated, corresponding to the clinical conditions. This comparative design enabled the assessment of how algorithmic tool selection influences the sensitivity and specificity of the resulting metabolic reconstructions. Analyses were performed in MATLAB R2025a using the COBRA Toolbox. Transcriptomic integration was carried out with the exp2flux pipeline (Angarita-Rodríguez et al., 2024), which applies Gene–Protein–Reaction (GPR) rules to constrain reaction bounds according to condition-specific gene expression.

A functional homeostatic objective was defined to represent astrocyte metabolic maintenance, integrating ATP turnover, glutamate–glutamine cycling, and redox balance (GSH, NAD^+^, and lactate) as the primary systems-level readout of cellular homeostasis.

Flux Balance Analysis (FBA) and Flux Variability Analysis (FVA) were applied to evaluate metabolic activity and flexibility under each condition. Flux variability intervals were used to quantify metabolic plasticity; narrow ranges identified metabolic bottlenecks, whereas broader intervals indicated pathway redundancy (Orth et al., 2010). Comparative fluxomics was performed to evaluate the impact of deconvolution strategy and disease progression on four fundamental astrocytic metabolic axes: glutamate metabolism, glutathione synthesis, lactate dehydrogenase activity, and lactate transport.

### 2.10 External validation of astrocyte signatures derived from DECODER and CDSeq using single-nucleus RNA-seq pseudobulk profiles

To evaluate the biological relevance of astrocyte-associated signals inferred from bulk transcriptomic data, we performed an external validation using an independent single-nucleus RNA-seq (snRNA-seq) dataset derived from the entorhinal cortex, spanning control individuals and multiple stages of Alzheimer’s disease progression (Serrano-Pozo et al., 2024). Astrocyte nuclei were subsetted from the reference dataset and aggregated into pseudobulk expression profiles using a donor-aware strategy, in which gene expression values were first averaged at the individual level and subsequently summarized across disease stages to reduce biases arising from unequal cell representation and sampling depth (Crowell et al., 2020).

To enable direct comparability between datasets, both CDSeq- and decoder-derived gene expression profiles were restricted to a shared set of genes and transformed using log2 scaling to stabilize variance and mitigate platform-specific effects (Wang et al., 2018). Given the cross-platform nature of the comparison (bulk hippocampal transcriptomic profiles versus brain-derived snRNA-seq), this normalization step was critical to ensure robust downstream analyses. The datasets and reference resources used for this validation, including their differences in brain region, sequencing platform, and disease stage, are summarized in Supplementary Table S1.

A targeted validation strategy was implemented using a curated set of astrocyte marker genes derived from established cell-type annotation resources (Brugulat-serrat et al., 2023), including canonical markers such as GFAP, AQP4, ALDH1L1, SLC1A2, and GJA1. This marker-based approach enables a biologically informed assessment of cell-type specificity while minimizing the influence of global transcriptomic variation.

Concordance between inferred and reference astrocyte profiles was quantified through stage-wise Pearson correlation analyses, as well as cross-stage correlation matrices to assess preservation of disease progression structure. This framework provides a robust benchmark for evaluating deconvolution performance against single-cell-derived ground truth (Avila Cobos et al., 2018).

Given the cross-platform (microarray vs. RNA-seq), cross-region (hippocampus vs. entorhinal cortex), and cross-modality (bulk vs. single-nucleus) nature of this validation framework, moderate correlation values are expected and should not be interpreted as lack of biological signal, but rather as attenuation due to technical and biological heterogeneity.

**FIGURE 1:**
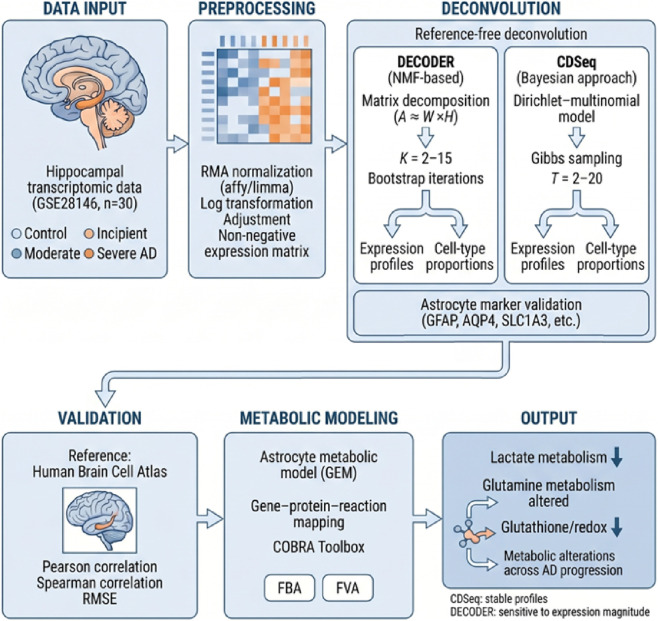
Overview of the integrative methodological framework for astrocyte-specific transcriptomic deconvolution and metabolic analysis. The workflow comprises four main stages: (i) data preprocessing, including normalization of hippocampal microarray data (GSE28146) and transformation into non-negative expression values; (ii) unsupervised transcriptomic deconvolution using DECODER and CDSeq to infer latent expression profiles and cell-type proportions across clinical conditions; (iii) validation of inferred astrocyte signatures through comparison with single-cell and single-nucleus RNA-seq reference datasets, including correlation analyses, gene overlap assessment, and enrichment of canonical astrocyte markers; and (iv) integration of deconvolution-derived profiles into a genome-scale metabolic model (GEM) of human astrocytes to simulate condition-specific metabolic fluxes using flux balance analysis (FBA) and flux variability analysis (FVA). This framework enables the systematic characterization of astrocyte transcriptional and metabolic alterations across the Alzheimer’s disease continuum.

An overview of the complete analytical workflow, including data preprocessing, deconvolution, validation, and metabolic integration, is summarized in Figure 1.

## 3 Results and discussion

The principal deconvolution outputs are provided both as Supplementary Material and through the public GitHub repository.

Astrocytes play a central role in brain homeostasis, metabolic regulation, neurovascular coupling, and neuroinflammatory responses. These homeostatic functions undergo progressive decline across the clinical continuum of Alzheimer’s disease (AD), from incipient impairment to severe neurodegeneration (Chen Y., 2022; Guebel, 2023).

Transcriptomic profiling enables the characterization of functional transitions between homeostatic and reactive astrocyte states. These transitions, which are characteristic of MCI, involve metabolic reprogramming and coordinated molecular, morphological, and functional changes (Counts et al., 2014; Fornazzari, 2001; Mufson et al., 2012).

In this study, we identified astrocyte-specific transcriptomic signatures as high-resolution indicators of clinical progression across the AD spectrum. Such changes reflect increased astrocytic reactivity to cellular stressors, including oxidative stress, and are associated with disruptions in key pathways such as glutamatergic synaptic regulation (Guebel, 2023; Luquez et al., 2022).

### 3.1 Network-based prioritization identifies CDSeq and DECODER as complementary reference-free deconvolution strategies

The heterogeneous cellular composition of the human brain represents a major challenge for the interpretation of bulk transcriptomic datasets, particularly in neurodegenerative disorders characterized by dynamic cellular remodeling and disease-associated transcriptional states (Luquez et al., 2022). Under these conditions, deconvolution methods provide an essential strategy to disentangle latent biological signals that would otherwise remain obscured within bulk tissue measurements. However, the large number of available algorithms and their distinct methodological assumptions complicate the selection of appropriate tools for downstream systems biology applications.

To address this challenge, we implemented a network-based prioritization framework integrating methodological, computational, and biological criteria. The framework incorporated 15 weighted feature categories including tissue heterogeneity, reference dependency, deconvolution strategy, computational efficiency, code availability, compatibility with RNA-seq and microarray data, and suitability for downstream metabolic modeling. Rather than focusing exclusively on deconvolution accuracy, the framework was designed to identify methods capable of recovering biologically meaningful transcriptomic compartments while remaining compatible with downstream functional analyses.

A total of 35 transcriptomic deconvolution tools were systematically evaluated through a weighted bipartite network framework integrating methodological features, implementation characteristics, scalability, robustness to tissue heterogeneity, reference dependency, and suitability for downstream metabolic modeling (Supplementary Figure S1; Supplementary Table S2–S4, S6). Network topology analysis enabled the identification of highly connected methods occupying central positions within the methodological landscape of transcriptomic deconvolution. Quantitative ranking revealed CDSeq as the highest-scoring method (score = 51), followed by DECODER (score = 48), DeconICA (score = 47), and TOAST (score = 45), supporting the prioritization of CDSeq and DECODER for downstream analyses (Supplementary Table S6).

Among the evaluated approaches, CDSeq and DECODER emerged as the two highest-priority reference-free strategies. Although both methods operate in an unsupervised framework, they rely on fundamentally different mathematical principles. DECODER employs a deterministic matrix factorization strategy based on non-negative matrix factorization and non-negative least squares (Peng et al., 2019), whereas CDSeq uses a probabilistic Bayesian framework based on Dirichlet distributions and Gibbs sampling (Kang et al., 2021). This methodological complementarity made them particularly suitable candidates for benchmarking biological signal recovery under heterogeneous neurodegenerative conditions.

To further characterize the biological structure recovered by the selected framework, DECODER was applied globally across all hippocampal samples, generating a hierarchical organization of latent transcriptomic compartments (Supplementary Figure S2A). The resulting factor tree revealed multiple stable and biologically interpretable modules associated with GABAergic signaling, calcium homeostasis, protein quality control, translational regulation, detoxification, and cellular stress responses. Several of these modules remained stable across factorization levels, supporting the ability of reference-free deconvolution approaches to recover reproducible biological programs from heterogeneous hippocampal tissue without relying on predefined cellular references.

Based on this prioritization strategy, CDSeq and DECODER were selected for downstream implementation and comparative evaluation. Subsequent analyses focused on determining whether statistically recovered transcriptomic compartments preserve biologically coherent signals and whether these signals remain functionally interpretable following integration into genome-scale metabolic models. Importantly, the objective of this prioritization strategy was not to identify a single best-performing algorithm, but rather to select complementary deconvolution approaches whose outputs could be independently evaluated through biological validation and functional metabolic analyses.

### 3.2 Reconstruction of condition-specific transcriptomic compartments

To enable direct comparison between deconvolution strategies and disease stages, transcriptomic compartments inferred by CDSeq and DECODER were transformed into condition-specific pseudobulk profiles (Supplementary Figure S2B). This reconstruction framework generated a common analytical representation that preserved the latent biological structure recovered by each algorithm while enabling downstream biological, statistical, and metabolic comparisons across the Alzheimer’s disease continuum.

For DECODER, transcriptomic compartments were first inferred globally across all samples and subsequently projected back into gene-by-sample expression space through the normalized multiplication of gene weight (gene_w) and sample weight (sample_w) matrices. The reconstructed expression matrix was then stratified according to clinical condition and averaged to generate four condition-specific pseudobulk profiles corresponding to Control, Incipient, Moderate, and Severe stages (Supplementary Figure S2B).

In contrast, CDSeq directly generated condition-specific transcriptomic profiles through independent probabilistic decomposition of each clinical cohort. This approach preserved local transcriptional structures within each disease stage and enabled the recovery of dynamic biological programs without imposing a common latent architecture across all samples.

### 3.3 Biological characterization of inferred transcriptomic compartments

Following transcriptomic reconstruction, we evaluated whether the latent compartments recovered by each deconvolution strategy captured biologically coherent signals associated with Alzheimer’s disease progression. Particular attention was given to the preservation of disease-relevant functional programs, stress-response pathways, and astrocyte-associated transcriptional dynamics.

**FIGURE 2:**
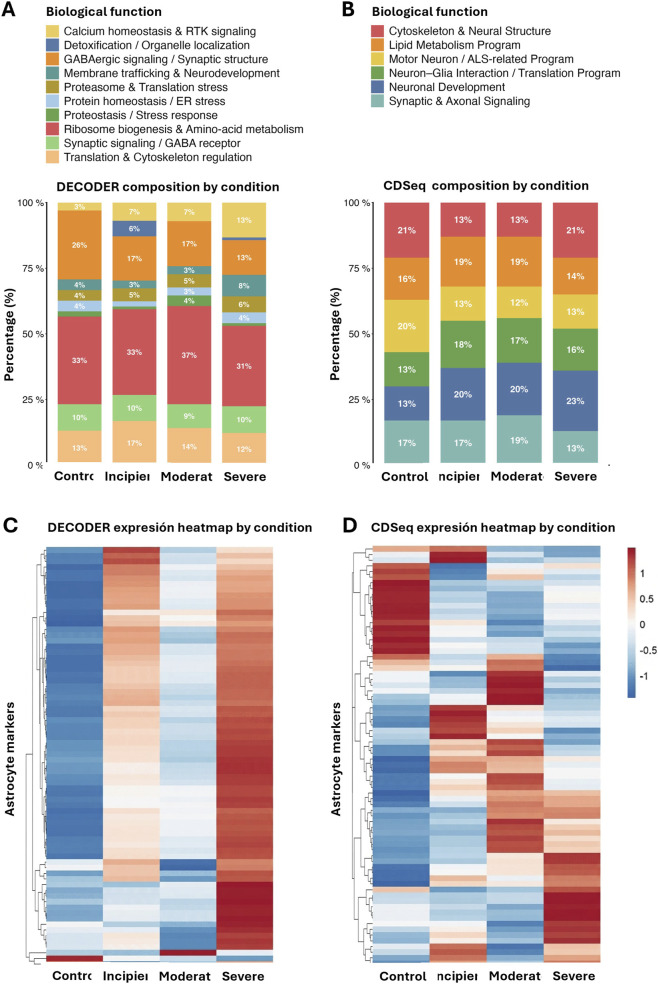
Transcriptomic alterations associated with clinical progression estimated by deconvolution tools. DECODER **(A,C)** and CDSeq **(B,D)** were used to infer transcriptomic profiles and expression dynamics across control, incipient, moderate, and severe stages of cognitive impairment. **(A)** Functional enrichment analysis of DECODER-derived latent compartments showing condition-specific activation of biological pathways associated with synaptic signaling, stress response, proteostasis, and translational regulation. **(B)** Relative proportions of CDSeq-derived latent components across clinical conditions, illustrating stable preservation of inferred transcriptomic structures throughout disease progression. **(C)** Heatmap of stress-associated and astrocyte-related genes identified within DECODER-derived compartments, highlighting progressive increases in genes related to proteostasis and cellular stress responses, including CRYAB and HSPA2. **(D)** Heatmap of canonical astrocyte markers within CDSeq-derived profiles, showing gradual upregulation of reactive astrocyte-associated genes such as GFAP, AQP4, ID1, ID3, and GSN across disease stages. Together, these results illustrate the distinct methodological behavior of both deconvolution approaches, with DECODER emphasizing broader transcriptional transitions and CDSeq preserving more stable gene–gene relationships across inferred astrocyte-associated compartments.

DECODER decomposes bulk transcriptomic signals into latent compartments, capturing state-specific gene programs that reflect functional astrocyte transitions. Functional enrichment analysis revealed condition-specific activation of biological processes (Figure 2A). Notably, pathways related to GABAergic synaptic signaling and synaptic structure exhibited a progressive reduction in receptor-mediated processes across clinical stages, consistent with the reported loss of inhibitory tone during aging and mild cognitive impairment. This trend was observed across the transition from Control to Severe AD compartments and was consistently recovered among the most stable DECODER factors.

In parallel, stress response pathways, including proteasome activity and translational stress, including modules associated with protein homeostasis, endoplasmic reticulum stress, and detoxification pathways, showed increased enrichment across conditions. These changes were accompanied by higher expression of stress-associated genes such as CRYAB and HSPA2 among the most representative stress-associated genes, which have been previously reported to be upregulated in astrocytes from Alzheimer’s disease samples (Figure 2C; Supplementary Figure S2A; detailed gene lists are provided in Supplementary Table S5).

These transcriptomic compartments ranked among the most stable factors identified by DECODER (Supplementary Figure S2A). Statistical significance of pathway enrichment was assessed using adjusted p-values, and only significantly enriched pathways were retained for downstream interpretation.

A complementary biological pattern emerged from CDSeq-derived profiles. Unlike DECODER, which emphasizes globally stable transcriptional compartments, CDSeq preserved condition-dependent transcriptional variation and enabled the identification of progressive molecular changes associated with disease severity. CDSeq demonstrated higher stability in inferred component proportions across clinical conditions (Figure 2B), leveraging Bayesian regularization to preserve relative gene–gene relationships. Therefore, downstream analyses focused on identifying biologically coherent astrocyte-associated compartments rather than assuming complete reconstruction of purified astrocyte transcriptomes.

Heatmap analysis of astrocyte marker genes revealed subtle but consistent patterns of progressive regulation across disease stages (Figure 2D; Supplementary Table S7). Genes such as GFAP, AQP4, ID3, ID1, and GSN showed increased expression, consistent with astrocytic reactivity, cytoskeletal remodeling, and altered water homeostasis previously associated with AD-related astrocyte activation.

Cell-type specificity analysis further supported the astrocytic identity of the selected compartments. Both methods showed enrichment of astrocyte-associated markers; however, CDSeq-derived profiles exhibited stronger astrocyte specificity and reduced contribution from neuronal, microglial, and oligodendroglial signatures compared with DECODER (Supplementary Figure S3).

Together, these results indicate that both deconvolution approaches recovered biologically interpretable transcriptomic programs associated with Alzheimer’s disease progression. DECODER highlighted broad functional transitions involving synaptic signaling, proteostasis, and cellular stress responses, whereas CDSeq preserved condition-dependent transcriptional structure, progressive astrocyte-associated marker dynamics, and greater astrocyte-specific enrichment relative to other major brain cell populations. These complementary patterns support the use of both methods for downstream biological validation, while emphasizing that inferred compartments should be interpreted as latent biological programs rather than purified cell-type transcriptomes.

### 3.4 Cell type–specific transcriptional dynamics across disease progression

**FIGURE 3:**
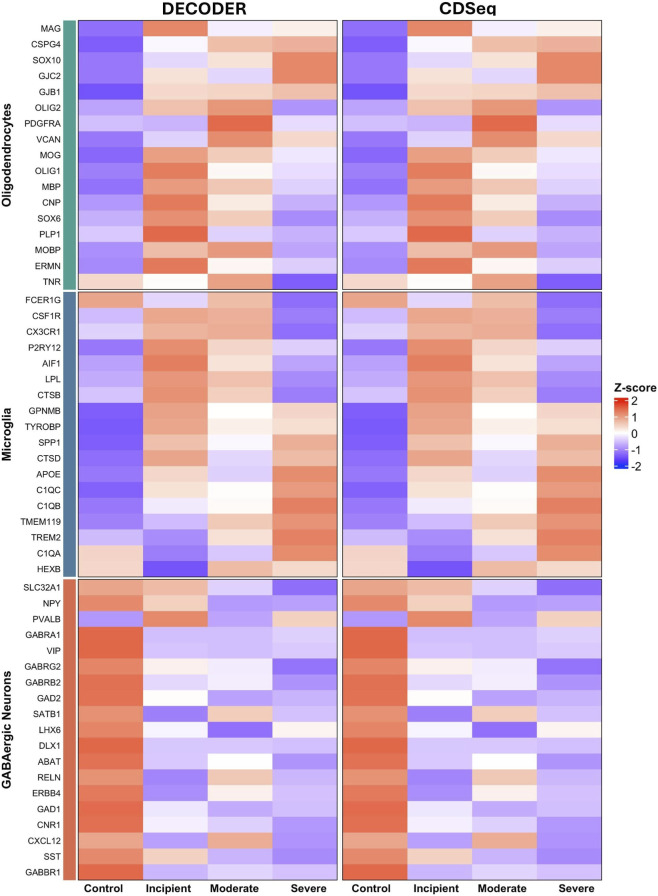
Cell type–specific marker expression across Alzheimer’s disease progression inferred by CDSeq and DECODER deconvolution methods. Heatmaps show normalized expression levels (Z-scores) of canonical marker genes for oligodendrocytes, microglia, and GABAergic neurons across four disease stages (Control, Incipient, Moderate, and Severe AD). Rows represent cell type–specific marker genes, and columns correspond to disease stages. Warmer colors (red) indicate relatively higher expression, whereas cooler colors (blue) indicate lower expression. The left and right panels display the expression patterns inferred by the two independent deconvolution approaches, CDSeq and DECODER, respectively. Oligodendrocyte markers exhibit increased expression at early and intermediate stages, with partial decline in severe disease. Microglial markers show elevated expression during Incipient and Moderate stages, consistent with progressive neuroinflammatory activation. In contrast, GABAergic neuronal markers display a gradual reduction across disease progression, particularly in Moderate and Severe stages, suggesting progressive neuronal dysfunction and loss. Overall, both deconvolution methods reveal highly concordant cell type–specific transcriptional changes associated with Alzheimer’s disease progression.

To determine whether the inferred transcriptomic compartments captured biologically meaningful multicellular responses beyond astrocyte-associated programs, we evaluated the expression of canonical marker genes representative of oligodendrocytes, microglia, and GABAergic neurons across disease progression (Figure 3). These observations are consistent with the cell-type specificity analysis shown in Supplementary Figure S3, which confirmed the enrichment of lineage-associated marker signatures in the selected deconvolved compartments.

Both deconvolution approaches recovered highly broadly consistent cell type–specific transcriptional trajectories across disease progression. Oligodendrocyte-associated markers showed increased expression during the Incipient and Moderate stages, followed by partial attenuation in Severe AD. Microglial markers exhibited elevated expression during Incipient and Moderate stages, consistent with progressive neuroinflammatory activation. In contrast, GABAergic neuronal markers displayed a gradual reduction across disease progression, particularly in Moderate and Severe stages, suggesting progressive impairment of inhibitory neuronal function and synaptic integrity.

Importantly, these patterns were independently recovered by both CDSeq and DECODER (Figure 3), supporting the notion that deconvolution-derived pseudobulks preserve disease-relevant cellular dynamics beyond astrocyte-associated programs.

### 3.5 Cell type–specific functional enrichment reveals coordinated biological remodeling

**FIGURE 4:**
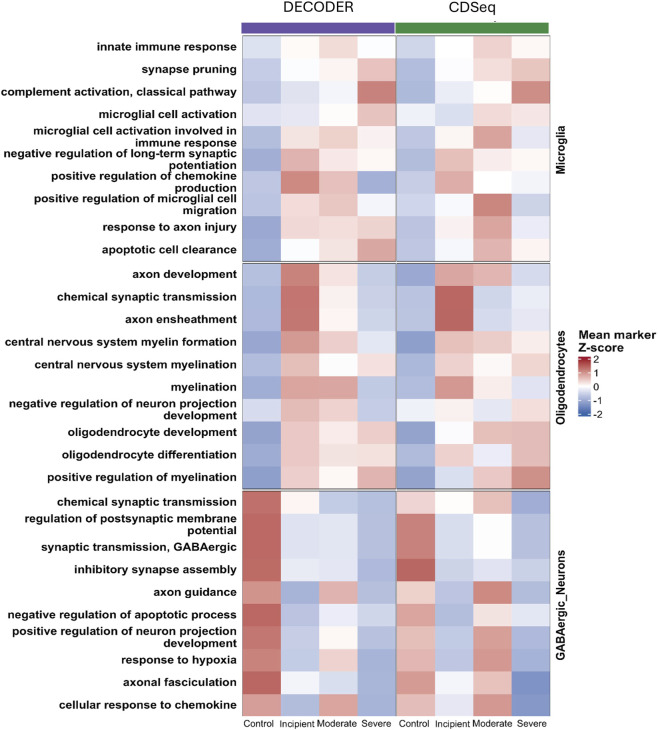
Cell type–specific Gene Ontology (GO) enrichment across Alzheimer’s disease progression inferred by DECODER and CDSeq. Heatmaps display the mean marker Z-scores of significantly enriched biological processes associated with microglia, oligodendrocytes, and GABAergic neurons across four disease stages (Control, Incipient, Moderate, and Severe AD). Biological processes were selected based on functional enrichment analyses of cell type–specific marker genes identified by the DECODER and CDSeq deconvolution approaches. Warmer colors (red) indicate stronger enrichment, whereas cooler colors (blue) indicate weaker enrichment relative to the overall dataset. Microglial signatures showed progressive enrichment of immune-related processes, including innate immune response, complement activation, microglial activation, chemokine production, and apoptotic cell clearance, particularly in Moderate and Severe stages, consistent with increasing neuroinflammatory activity. Oligodendrocyte-associated pathways were enriched for myelination, axon ensheathment, oligodendrocyte differentiation, and axon development, with strongest enrichment observed during the Incipient and Moderate stages. In contrast, GABAergic neuron-associated processes, including synaptic transmission, inhibitory synapse assembly, axon guidance, and neuronal projection development, exhibited the highest enrichment in Controls and progressively declined across disease stages, indicating progressive impairment of neuronal connectivity and inhibitory neurotransmission. Overall, both deconvolution methods revealed highly concordant functional alterations across cell populations during Alzheimer’s disease progression.

To further evaluate the functional relevance of the recovered cellular programs, Gene Ontology enrichment analyses were performed using marker genes associated with each major cell population (Figure 4).

Microglial-associated signatures exhibited progressive enrichment of immune-related biological processes, including innate immune response, complement activation, chemokine production, apoptotic cell clearance, and microglial activation. These immune-related enrichments were most pronounced during the Moderate and Severe stages of disease progression (Figure 4).

Oligodendrocyte-associated programs were enriched for myelination, axon ensheathment, oligodendrocyte differentiation, and axonal development, with strongest enrichment observed during Incipient and Moderate stages. This pattern mirrors the transcriptional dynamics observed in Figure 3, suggesting an early compensatory response involving myelin maintenance and axonal support. In contrast, GABAergic neuron–associated processes, including synaptic transmission, inhibitory synapse assembly, neuronal projection development, and axon guidance, progressively declined across disease stages (Figure 4), consistent with the reduction of GABAergic signaling pathways identified in DECODER-derived compartments (Figure 2A).

The overall consistency observed between CDSeq and DECODER indicates that both approaches recover biologically meaningful functional programs. Collectively, these results support the interpretation that the inferred transcriptomic compartments reflect coordinated multicellular remodeling rather than isolated astrocyte-specific responses. Together, these results indicate that disease progression is accompanied by coordinated remodeling of immune, myelin-associated, and synaptic programs across multiple brain cell populations.

### 3.6 Validation of astrocyte-associated compartments

To ensure that the inferred transcriptomic compartments corresponded to astrocyte-associated biological signals rather than generic stress responses, we implemented a multi-level validation strategy integrating canonical marker enrichment, cell-type specificity analyses, and functional coherence assessments.

Importantly, the transcriptomic compartments inferred by CDSeq and DECODER should not be interpreted as pure cell-type–specific profiles. Due to the unsupervised nature of both approaches, the recovered components represent latent biological programs that may partially capture transcriptional signals shared across multiple brain cell populations. Therefore, the biological interpretation of these compartments relies on convergent evidence from marker enrichment, functional characterization, and external validation rather than on the assumption of discrete cellular identity.

First, we evaluated the expression of canonical astrocyte markers, including canonical astrocyte markers such as GFAP, AQP4, ALDH1L1, SLC1A2, SLC1A3, GJA1, GLUL, FGFR3, and S100B. Astrocyte-associated compartments consistently displayed elevated expression of these markers relative to other inferred components (Figure 2D; Supplementary Figure S3), supporting their biological relevance. Furthermore, CDSeq-derived profiles exhibited structured and stage-dependent transcriptional variation across the Alzheimer’s disease continuum, whereas DECODER-derived profiles displayed comparatively homogeneous expression patterns (Figure 2D). This observation suggests that CDSeq more effectively preserves condition-specific transcriptional dynamics, while DECODER prioritizes reconstruction of global expression magnitude. Consistent with this observation, CDSeq-derived profiles also showed stronger astrocyte-specific enrichment and reduced contribution from neuronal, microglial, and oligodendroglial signatures compared with DECODER (Supplementary Figure S3), further supporting the biological relevance of the selected astrocyte-associated compartments.

The progressive upregulation of genes such as GFAP and AQP4 observed in CDSeq-derived compartments is consistent with the emergence of reactive astrocyte phenotypes previously described in Alzheimer’s disease and supports the biological plausibility of the inferred astrocyte-associated programs.

To further assess cell-type specificity, marker enrichment scores were calculated for major brain cell populations, including astrocytes, neurons, microglia, and oligodendrocytes (Supplementary Figure S3). CDSeq-derived profiles showed strong and consistent enrichment of astrocyte markers across all clinical stages, accompanied by relatively low enrichment of neuronal, microglial, and oligodendrocyte signatures. In contrast, DECODER-derived profiles exhibited a more heterogeneous pattern, with detectable contributions from multiple cell types. These results indicate that CDSeq preserves a higher degree of astrocyte specificity while reducing potential signal mixing between cellular populations.

Importantly, this specificity analysis complements the multicellular patterns identified in Figures 3, 4. While both deconvolution approaches successfully recovered disease-associated transcriptional trajectories involving microglia, oligodendrocytes, and GABAergic neurons, CDSeq more consistently preserved astrocyte-associated transcriptional programs, supporting its suitability for downstream astrocyte-centered analyses.

Finally, functional characterization provided additional evidence supporting biological specificity. Astrocyte-associated compartments showed enrichment of pathways related to glutamate uptake, lactate transport, redox homeostasis, and metabolic support functions, all of which represent core astrocytic processes involved in neuronal maintenance and neuroprotection. The convergence of marker enrichment, cell-type specificity, and functional coherence supports the interpretation that the inferred compartments capture biologically meaningful astrocyte-associated programs rather than generic transcriptional responses.

To further assess whether these astrocyte-associated compartments recapitulate independent astrocyte transcriptional signatures, we next compared deconvolved profiles against external single-nucleus RNA-seq–derived astrocyte pseudobulk references.

### 3.7 Statistical and concordance analysis between tools

Various statistical analyses were performed to evaluate concordance between deconvolution-derived pseudobulks and the astrocyte reference pseudobulk across clinical conditions. Comparative metrics revealed distinct performance profiles between both methods (Tables 1 and 2). DECODER exhibited lower RMSE values and closer approximation to absolute expression magnitudes, whereas CDSeq demonstrated greater preservation of relative gene–gene relationships and more stable concordance across disease stages.

**TABLE 1:** Statistical analysis of the accuracy of transcriptomic deconvolution tools against the reference profile (astrocytes from the Human Brain Cell Atlas v1.0). Pearson and Spearman correlations, and root mean square error (RMSE) between control pseudobulks estimated by CDSeq and DECODER and the reference profile. DECODER showed higher concordance with reference expression magnitudes and lower reconstruction error than CDSeq.

Tool	Pearson correlation	Spearman correlation	RMSE
DECODER	0.387	0.613	0.0001104
CDSeq	0.196	0.542	0.0003478

**TABLE 2:** Concordance and robustness metrics of pseudobulks generated by DECODER and CDSeq. Pearson and Spearman correlations, and root mean square error (RMSE) between pseudobulks estimated across four clinical conditions (Control, Early, Moderate, and Severe), as well as robustness analysis of the pseudobulks following Hu and Chikina (2024).

Condition	Pearson correlation	Spearman correlation	RMSE
Control	0.343	0.887	0.000355
Incipient	0.340	0.897	0.000358
Moderate	0.347	0.896	0.000345
Severe	0.345	0.895	0.000352
Pseudobulk*	0.204	0.970	0.000778

*Robustness Analysis Result.

Pearson and Spearman correlation analyses showed moderate but consistent agreement between inferred profiles and astrocyte reference signatures, supporting the recovery of biologically coherent astrocyte-associated transcriptional programs despite substantial technical and biological heterogeneity between datasets. Detailed concordance metrics, including correlation coefficients and RMSE values across clinical conditions, are summarized in Tables 1 and 2.

#### 3.7.1 Accuracy assessment and gene overlap

**FIGURE 5:**
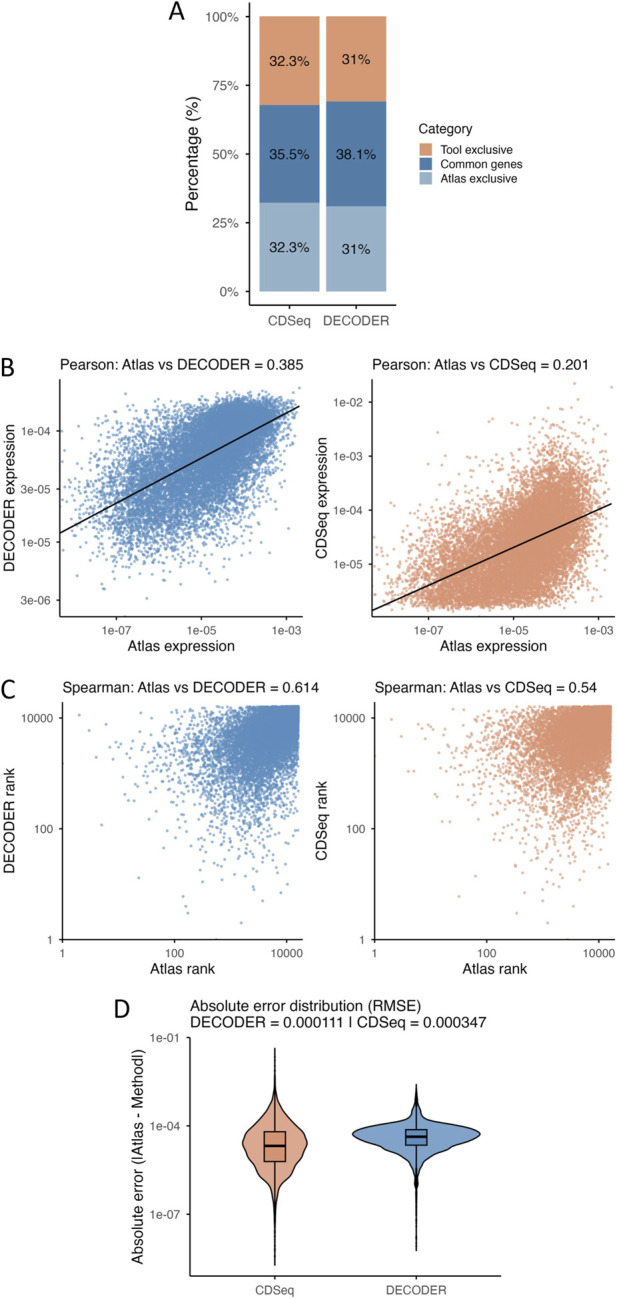
Accuracy assessment and gene-level concordance between deconvolution-derived pseudobulks and single-cell astrocyte reference profiles. **(A)** Overlap among the top 30% most highly expressed genes in the control condition, showing shared and method-specific gene sets for CDSeq and DECODER relative to the reference atlas. **(B)** Scatter plot comparing predicted versus reference gene expression values, illustrating closer approximation to the identity line for DECODER and wider dispersion for CDSeq. **(C)** Distribution of gene expression intensities, highlighting a more homogeneous profile for DECODER-derived pseudobulks. **(D)** Distribution of absolute errors (RMSE), indicating lower variability for DECODER. Together, these analyses demonstrate partial reconstruction of astrocyte-specific transcriptional signals, with DECODER showing improved preservation of linear expression structure.

Comparison of the overlap among the top 30% most highly expressed genes in the control condition showed that both CDSeq (35.6%) and DECODER (38.2%) recovered a subset of genes shared with the single-cell reference atlas (Figure 5A). Notably, a substantial fraction of highly expressed genes remained exclusive to each dataset, reflecting intrinsic differences between bulk and single-cell transcriptomic profiles.

This pattern is further illustrated in Figure 5B, where DECODER predictions show a closer approximation to the identity line, whereas CDSeq exhibits a wider dispersion. Consistently, Figure 5C shows a more homogeneous distribution of expression intensities for DECODER, while Figure 5D highlights lower variability in absolute errors (RMSE).

Although single-cell RNA-seq data are affected by sparsity and technical dropout, which may limit direct comparability with bulk-derived profiles, the moderate overlap observed here is unlikely to be solely explained by zero-inflation effects. Instead, it likely reflects differences in data structure, normalization strategies, and the inherent complexity of reconstructing cell-type–specific signals from bulk mixtures.

Tool-versus-atlas comparisons revealed distinct performance profiles. DECODER achieved higher concordance with the astrocyte reference profile, with Pearson and Spearman correlations of 0.389 and 0.613, respectively, compared with 0.195 and 0.542 for CDSeq. DECODER also showed a lower reconstruction error (RMSE = 1.10 × 10^−4^) than CDSeq (RMSE = 3.48 × 10^−4^), indicating a closer approximation of reference expression magnitudes (Table 1). Although DECODER outperformed CDSeq according to all three metrics, the moderate correlation coefficients observed for both methods indicate only a partial reconstruction of the astrocyte-associated transcriptomic signal relative to the external reference.

Accordingly, these results support the interpretation that both methods capture an astrocyte-associated transcriptomic compartment rather than a fully resolved cell-type–specific expression profile. In this context, DECODER appears to better preserve the linear structure of gene expression, particularly for genes involved in core astrocytic functions such as energy metabolism and the glutamate–glutamine cycle. This distinction is particularly relevant in neurodegenerative tissues, where reactive glial programs and stress-response pathways may overlap across multiple cellular populations.

Overall, although concordance with the reference remains moderate, these findings suggest that DECODER provides a slightly improved approximation of reference expression magnitudes, whereas CDSeq more effectively preserves astrocyte-associated biological specificity and disease-stage transcriptional structure. DECODER showed higher agreement with reference expression magnitudes under this benchmarking framework, outperforming CDSeq in reconstructing global expression levels. However, CDSeq provides superior recovery of biologically meaningful astrocyte-specific signals, as supported by the cell-type specificity and validation analyses presented in this study (Figure 7).

These differences can be explained by the nature of the algorithms employed in each tool. DECODER uses an NMF scheme executed over multiple iterations, which favors reconstruction error minimization and component stabilization (Brunet et al., 2003; Lin and Boutros, 2020). This deterministic approach prioritizes quantitative fidelity, resulting in higher linear correlations with reference profiles. In contrast, CDSeq relies on a hierarchical Bayesian (Dirichlet–MCMC) model aimed at optimizing cell membership probabilities and proportions, which tends to preserve relative gene relationships more accurately but may reduce linear correlation with reference data (Erkkilä et al., 2010; Kang et al., 2021).

#### 3.7.2 Concordance and robustness analysis between algorithms

Methodological concordance between DECODER and CDSeq was assessed across all four clinical conditions using pseudobulks generated by each tool. The analysis evaluated whether both algorithms consistently capture gene activation and repression patterns associated with disease progression and astrocyte-associated transcriptional remodeling.

**FIGURE 6:**
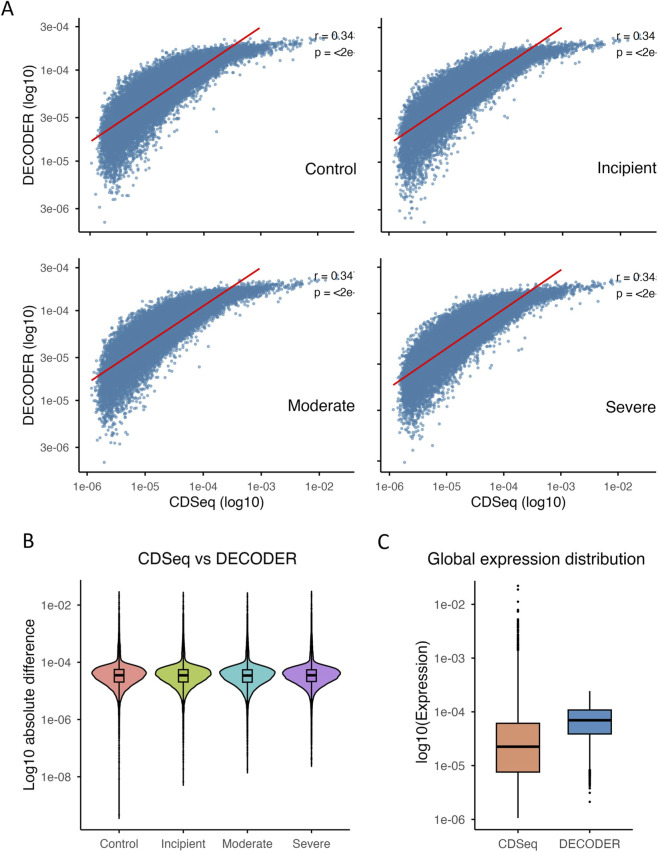
Concordance and robustness of pseudobulks estimated by DECODER and CDSeq. Both tools preserve global expression trends across clinical conditions. However, they differ in absolute expression magnitude. DECODER shows higher sensitivity, whereas CDSeq exhibits greater stability. **(A)** Comparison of mean expression values by clinical condition. The red line indicates the linear fit used for Pearson correlation. **(B)** Distribution of absolute expression differences between pseudobulks. **(C)** Robustness analysis based on Hu and Chikina (2024), showing the distribution of mean expression values for each tool (median, interquartile range Q1–Q3, and outliers). All panels are shown on a logarithmic scale to enhance visualization and emphasize structural differences.

Across clinical conditions, Spearman correlation coefficients remained high, indicating strong agreement in the relative ordering and direction of transcriptional changes inferred by both methods. In contrast, Pearson correlations were moderate, reflecting differences in absolute expression magnitudes that may arise from normalization procedures and model-specific scaling effects. Consistently, RMSE values remained low, suggesting that algorithmic differences were primarily driven by global signal rescaling rather than gene-specific divergence (Table 2; Figures 6A,B). Together, these results indicate that both tools preserve the overall transcriptomic structure associated with disease progression despite differences in absolute expression levels.

Importantly, while both methods produce concordant profiles across clinical conditions, these results do not support a full reconstruction of the global astrocyte transcriptome. Instead, they indicate that both approaches capture consistent transcriptomic patterns or compartments associated with disease progression.

To further assess robustness, an integrated pseudobulk was constructed from four major brain cell types (interneurons, pyramidal neurons, astrocytes, and microglia) following Hu and Chikina (2024). Results were consistent with the primary analysis: Spearman correlations remained high, Pearson correlations decreased, and RMSE values remained moderately low (Table 2; Figure 6C). Similar medians and interquartile ranges across methods suggest preservation of distributional structure and do not indicate strong dominance by any individual cell-type signature.

Overall, although Pearson correlations are attenuated by global magnitude rescaling, the combination of high Spearman correlations and low RMSE confirms that both algorithms preserve system-level transcriptomic structure. This concordance supports their suitability for downstream integration into genome-scale metabolic models (Hu and Chikina, 2024; Nguyen et al., 2024).

These findings demonstrate that the choice of deconvolution strategy influences both transcriptomic reconstruction and downstream biological interpretation. DECODER provides a closer approximation of global expression magnitudes, whereas CDSeq better preserves disease-stage transcriptional structure and astrocyte-associated biological specificity (Supplementary Figure S3).

### 3.8 Metabolic integration and functional analysis

To evaluate the functional impact of deconvolution-derived transcriptomic profiles, gene expression data were integrated into a curated human astrocyte metabolic model (Angarita-Rodríguez et al., 2024). Model integration was performed using the COBRA Toolbox, enabling systematic mapping of transcriptomic variation into metabolic constraints.

This approach translates gene expression changes into metabolic constraints, enabling the inference of functional effects from transcriptomic variation. Expression-to-reaction mapping allows gene dysregulation to be propagated to flux-level phenotypes, providing system-level resolution beyond cell-type proportion estimates.

Transcriptomic profiles generated by each deconvolution tool and clinical condition were mapped onto four functionally relevant astrocyte metabolic axes: (i) glutamate–glutamine cycling, (ii) lactate metabolism and transport, (iii) redox homeostasis mediated by glutathione, and (iv) cellular maintenance demands.

Metabolic fluxes were simulated using flux balance analysis (FBA) and flux variability analysis (FVA), with a composite homeostatic objective function integrating ATP turnover, the glutamate–glutamine cycle, and redox balance (Table 3).

**TABLE 3:** Metabolic fluxes (FBA) simulated in the astrocyte model (Angarita-Rodríguez et al., 2024) parameterized with CDSeq transcriptomic profiles. Metabolic fluxes (mM gDW^−1^ h^−1^) were calculated across the four clinical conditions, identifying five key metabolites related to biomass maintenance and astrocyte homeostasis.

Scenario	Metabolic Fluxes_DECODER (mMgWD^−1^ h^−1^)	Metabolic Fluxes_CDSEQ (mMgWD^−1^ h^−1^)
Experimental* 0.32 mM gDW^−1^ h^−1^		
Control	0.33	0.3304
Early MCI	0.30	0.3211
Advanced MCI	0.28	0.2718
AD	0.099	0.3037

*in vitro growth flux reported by (Prah et al., 2019).

Because transcriptomic inputs were derived from pseudocount-based profiles, results were interpreted as relative functional trends rather than absolute flux values. Accordingly, the analysis focused on comparative changes in flux ranges and metabolic flexibility between deconvolution methods across clinical conditions.

Accordingly, inferred metabolic states should be interpreted as system-level approximations of pathway activity and metabolic flexibility rather than direct measurements of enzymatic activity or intracellular flux. This framework does not explicitly account for post-translational regulation, enzyme kinetics, metabolite inhibition, or compartment-specific regulatory mechanisms, which may further influence astrocyte metabolism during Alzheimer’s disease progression.

**FIGURE 7:**
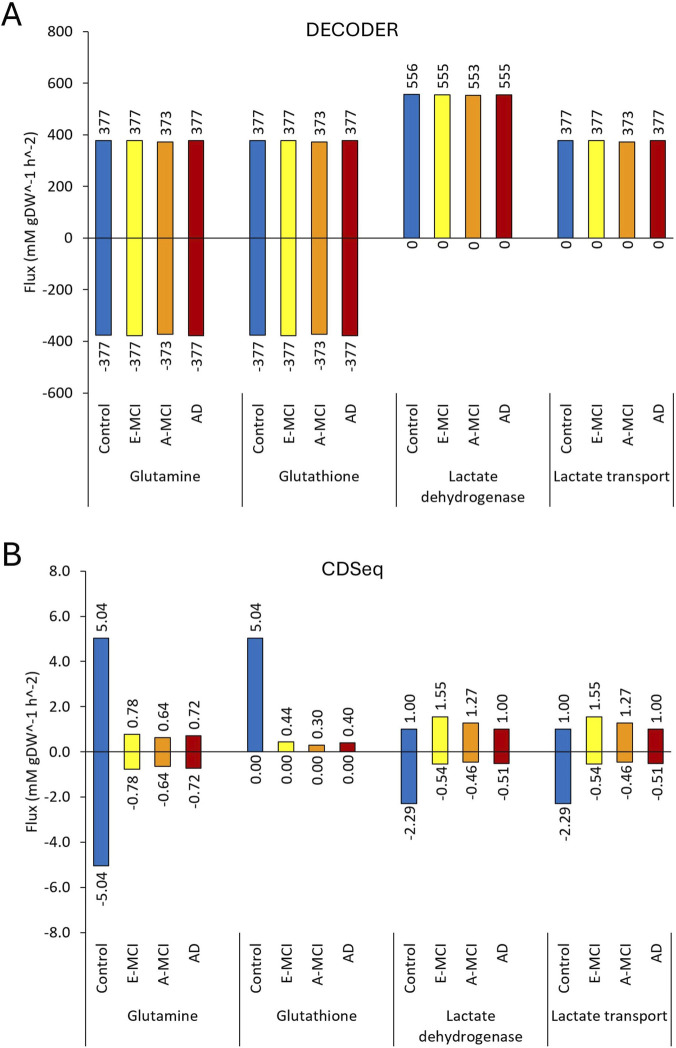
Comparative flux variability analysis of astrocyte-associated metabolic pathways inferred from DECODER and CDSeq transcriptomic profiles. Flux variability analysis (FVA) was performed on genome-scale astrocyte metabolic models constrained with deconvolution-derived transcriptomic profiles across Control, Early-MCI (E-MCI), Advanced-MCI (A-MCI), and Alzheimer’s disease (AD) conditions. Four metabolic processes associated with astrocyte function were evaluated: glutamine metabolism, glutathione-mediated redox homeostasis, lactate dehydrogenase activity, and lactate transport. **(A)** DECODER-derived metabolic models exhibited broad and relatively stable flux ranges across disease stages, suggesting preservation of metabolic flexibility despite disease progression. **(B)** CDSeq-derived metabolic models showed narrower and more condition-dependent flux distributions, indicating tighter metabolic constraints and increased sensitivity to disease-associated transcriptional alterations. Glutamine and glutathione metabolism displayed reduced metabolic flexibility under CDSeq-derived constraints, whereas lactate dehydrogenase activity and lactate transport showed stage-dependent alterations consistent with changes in astrocyte–neuron metabolic coupling. Together, these results demonstrate that the choice of deconvolution strategy influences downstream metabolic predictions, affecting the inferred balance between metabolic robustness, redox regulation, neurotransmitter recycling, and lactate-mediated energetic support during Alzheimer’s disease progression.

Flux variability analysis revealed distinct metabolic behaviors between deconvolution strategies across disease progression (Figure 7). Four astrocyte-associated metabolic axes were examined, including glutamine metabolism, glutathione-mediated redox homeostasis, lactate dehydrogenase activity, and lactate transport. Together, these pathways capture key aspects of neurotransmitter recycling, antioxidant defense, and astrocyte–neuron metabolic coupling. DECODER-derived metabolic models exhibited broad and relatively stable flux ranges across disease stages, suggesting preservation of metabolic flexibility despite disease progression. In contrast, CDSeq-derived models showed narrower and more condition-dependent flux distributions, indicating tighter metabolic constraints and greater sensitivity to disease-associated transcriptional alterations (Figure 7).

#### 3.8.1 Metabolic trends derived from CDSeq

The expression profiles derived from CDSeq produced relatively narrow FVA ranges and consistent FBA estimates across clinical conditions (Figure 7B), resulting in physiologically coherent flux trajectories. Notably, control samples exhibited comparatively broader FVA ranges, consistent with a more flexible metabolic state under homeostatic conditions, whereas disease states showed tighter flux constraints.

These results indicate a progressive reduction in metabolic flexibility across disease stages. Compared with controls, disease-associated models exhibited consistently narrower FVA intervals across glutamine, glutathione, lactate dehydrogenase, and lactate transport reactions (Figure 7B), suggesting increased metabolic constraint during disease progression.

In contrast to the broader and comparatively stable flux distributions observed in DECODER-derived models, CDSeq-derived constraints generated more stage-dependent metabolic responses across all four metabolic axes.

Lactate transport (LT) reactions showed a progressive reduction in flux variability across disease stages, with the feasible flux range decreasing from ±0.777 mM gDW^−1^ h^−1^ in Control samples to ±0.673 mM gDW^−1^ h^−1^ in Severe AD (Figure 7B). This progressive narrowing of the feasible flux range suggests reduced metabolic flexibility in astrocyte–neuron metabolic coupling under disease conditions. Similar impairments in lactate handling have been associated with decreased expression of monocarboxylate transporters (MCT1, MCT2, and MCT4) and reduced brain lactate levels in the APP/PS1 mouse model of AD (Zhang et al., 2018). Such alterations have been interpreted as evidence of partial disruption the astrocyte–neuron lactate shuttle, a mechanism that contributes to neuronal energy support and metabolic homeostasis (Bélanger and Magistretti, 2009; Magistretti and Allaman, 2018).

Lactate dehydrogenase (LDH) activity exhibited a transient expansion of its feasible flux range, increasing from 0 to 1.00 mM gDW^−1^ h^−1^ in Control samples to 0–1.55 mM gDW^−1^ h^−1^ in E-MCI, before returning to 0–1.00 mM gDW^−1^ h^−1^ in Severe AD (Figure 7B). This temporary increase may reflect a compensatory metabolic response aimed at maintaining lactate interconversion when transport capacity becomes progressively constrained. The subsequent normalization of LDH flux ranges in later disease stages suggests a gradual loss of this compensatory capacity as metabolic dysfunction advances.

Glutamine-associated fluxes exhibited the strongest disease-related reduction, with the feasible flux range decreasing from ±0.777 mM gDW^−1^ h^−1^ in Control samples to ±0.673 mM gDW^−1^ h^−1^ in Severe AD (Figure 7B). Given the central role of astrocytes in the glutamate–glutamine cycle, this reduction may reflect impaired neurotransmitter recycling and reduced metabolic support for neuronal function. Similarly, glutathione-associated fluxes became progressively constrained under disease conditions, with the upper flux bound decreasing from 0.777 mM gDW^−1^ h^−1^ in Control samples to 0.673 mM gDW^−1^ h^−1^ in Severe AD (Figure 7B). This pattern is consistent with reduced antioxidant buffering capacity and supports previous reports describing oxidative stress–associated astrocytic dysfunction in Alzheimer’s disease.

In this context, the observed increase in the upper bound of LDH flux ranges suggests a potential shift in metabolic balance toward lactate interconversion, consistent with a compensatory response to impaired lactate exchange. Similar dynamics were reported by (Zebhauser et al., 2022), who observed elevated lactate levels in early disease stages followed by a subsequent decline. Overall, these findings suggest an early increase in lactate metabolic activity, followed by a progressive functional decline.

Taken together, the reductions observed in glutamine metabolism, glutathione homeostasis, and lactate-associated pathways suggest coordinated impairment of neurotransmitter recycling, antioxidant defense, and astrocyte–neuron metabolic coupling. Such alterations are consistent with reports of disrupted redox homeostasis and astrocytic dysfunction under oxidative stress, where the uncoupling of energetic and antioxidant pathways constitutes a critical metabolic vulnerability that impairs astrocyte-mediated neuroprotection (Angarita-Rodríguez et al., 2024).

In conclusion, CDSeq-derived profiles generate a physiologically coherent astrocyte metabolic model that captured disease-associated changes in metabolic flexibility across clinical stages (Figure 7B). The progressive reduction in flux variability observed across glutamine, glutathione, and lactate-associated pathways supports the ability of CDSeq-derived constraints to capture disease-related metabolic alterations while preserving biologically interpretable reaction-level dynamics across clinical stages.

#### 3.8.2 Metabolic trends derived from DECODER

DECODER-inferred expression profiles yielded broader FVA and FBA ranges, reflecting a higher degree of metabolic plasticity in response to absolute expression magnitude shifts (Figure 7A).

Biomass maintenance showed a gradual reduction across disease stages, decreasing from 0.3462 mM gDW^−1^ h^−1^ in Control samples to 0.3211 mM gDW^−1^ h^−1^ in Severe AD (Figure 7A). This pattern is compatible with a progressive reduction in anabolic and biosynthetic capacity, consistent with energetic deterioration reported in advanced AD. In contrast to CDSeq-derived models, DECODER-derived metabolic models exhibited remarkably stable flux variability ranges across disease stages. Lactate transport reactions remained largely unchanged, varying only between ±377.31 and ±376.88 mM gDW^−1^ h^−1^ across conditions (Figure 7A). Similarly, lactate dehydrogenase (LDH) activity showed minimal variation, with upper flux bounds ranging from 553 to 556 mM gDW^−1^ h^−1^. These results suggest preservation of broad metabolic feasibility despite disease progression (Angarita-Rodríguez et al., 2024; Zhang et al., 2018).

Glutamine-associated fluxes also remained highly stable, fluctuating only between ±377.31 and ±376.88 mM gDW^−1^ h^−1^ across clinical stages (Figure 7A). Likewise, glutathione-associated fluxes exhibited consistently broad feasible ranges under all conditions. Unlike the constrained profiles observed in CDSeq-derived models, these results indicate that DECODER preserves substantial metabolic flexibility across disease stages. Such metabolic flexibility is consistent with the established role of astrocytes in maintaining energetic homeostasis and metabolic support for neuronal function (Magistretti and Allaman, 2015).

The stability observed across glutamine metabolism, glutathione homeostasis, and lactate-associated pathways suggests that DECODER primarily captures large-scale expression magnitude changes while maintaining broad feasible solution spaces. Consequently, disease-associated metabolic differences are represented primarily as shifts in overall metabolic capacity rather than pronounced pathway-specific constraints. This interpretation remains consistent with reports describing energetic dysfunction, oxidative stress, and altered astrocyte support functions during AD progression (Angarita-Rodríguez et al., 2024).

Both methods provide complementary insights for metabolic integration. CDSeq yields Dirichlet-regularized, stable profiles that preserve relative gene–gene relationships and produces comparatively narrow FVA ranges (Figure 7B). These features facilitate the identification of progressive metabolic trends with lower stochastic variability (Hu and Chikina, 2024; Kang et al., 2021).

DECODER, by contrast, is more sensitive to absolute expression changes and preserves broad feasible metabolic solution spaces (Figure 7A). While this approach reproduces expression magnitudes more directly, it generates wider FVA ranges and consequently emphasizes global metabolic capacity rather than disease-specific pathway constraints. The divergence between methods therefore reflects system-level signal rescaling rather than contradictions at the level of individual genes or reactions.

Given the distinct transcriptomic and metabolic behaviors recovered by CDSeq and DECODER, external validation was performed to determine which deconvolution strategy more accurately recapitulates independent astrocyte-specific transcriptional signatures.

### 3.9 External validation of astrocyte signatures derived from DECODER and CDSeq

External validation revealed detectable differences in concordance between methods, highlighting variability in the extent to which each approach captures cell-type–specific transcriptional profiles. While overall agreement was moderate, these differences underscore the influence of methodological assumptions and reference frameworks on deconvolution outcomes. Importantly, despite this variability, CDSeq-derived signatures consistently preserved key astrocytic features at both the gene and pathway levels. These findings provide a computational framework for investigating astrocyte-associated transcriptional and metabolic alterations in Alzheimer’s disease, supporting the use of deconvolution-based strategies to explore cell-type–specific mechanisms in complex neurodegenerative contexts.

**FIGURE 8:**
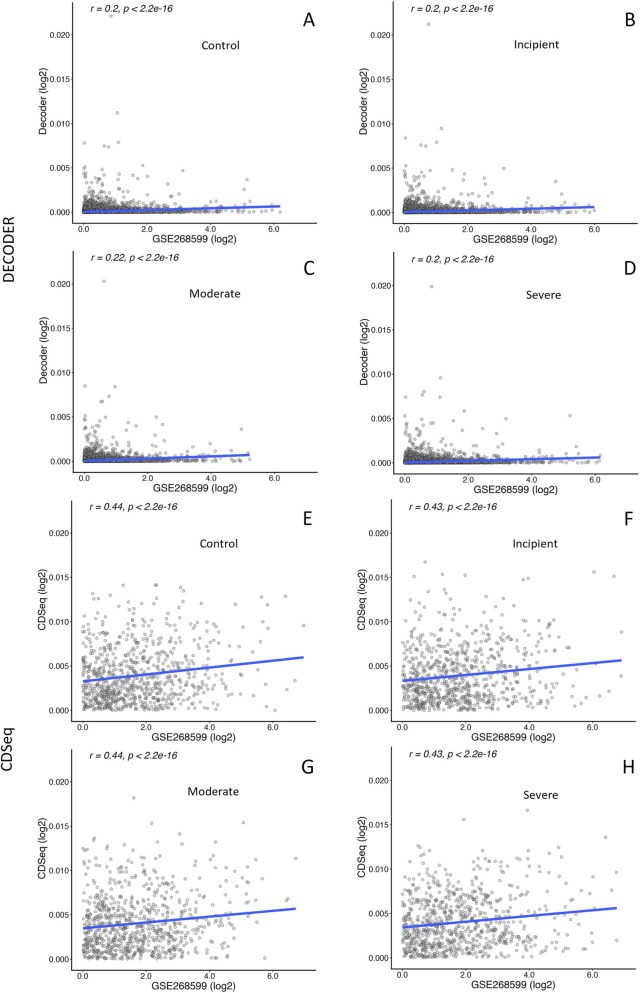
External validation of astrocyte-associated transcriptomic profiles inferred by DECODER and CDSeq using an independent single-nucleus RNA-seq dataset (SEA-AD). **(A–D)** Scatter plots showing the correlation between astrocyte gene expression profiles inferred by DECODER and independent SEA-AD single-nucleus RNA-seq dataset for the control **(A)**, Incipient **(B)**, Moderate **(C)**, and Severe **(D)** clinical conditions. **(E–H)** Scatter plots showing the correlation between astrocyte gene expression profiles inferred by CDSeq and independent SEA-AD dataset for the control **(E)**, Incipient **(F)**, Moderate **(G)**, and Severe **(H)** clinical conditions. Each point represents a shared gene between the deconvolved transcriptomic profile and the independent astrocyte reference. Pearson correlation coefficients (r) and associated p-values are indicated in each panel. These analyses demonstrate the preservation of astrocyte-associated transcriptional programs across disease stages and provide external biological validation of the inferred transcriptomic profiles.

Stage-wise correlation analysis demonstrated that CDSeq-derived profiles exhibit consistent and moderate concordance with the snRNA-seq astrocyte reference across all disease stages, with Pearson correlation coefficients of r = 0.44 (Control), 0.43 (Incipient), 0.44 (Moderate), and 0.43 (Severe) (p < 2.2 × 10^−16^) (Figures 8E–H). Although moderate in magnitude, these correlations should be interpreted as biologically meaningful and robust given the cross-platform and cross-region nature of the comparison, which integrates deconvolved bulk microarray-derived transcriptomic profiles with directly measured single-nucleus RNA-seq astrocyte references obtained from a distinct brain region. Under these conditions, attenuation of global correlation values is expected due to substantial technical and biological heterogeneity across platforms, tissues, and measurement modalities (Avila Cobos et al., 2020; Wang et al., 2018).

Crucially, this analysis highlights that biological validation should not rely solely on correlation magnitude but must also consider gene-level conservation and pathway-level enrichment. In this framework, the combination of moderate yet consistent correlations, preservation of canonical astrocytic markers, and enrichment of astrocyte-specific pathways provide strong support for the validity of CDSeq-derived astrocyte signatures. Altogether, these findings indicate that the deconvolution approach captures a coherent and biologically plausible astrocyte-associated transcriptional signal, reinforcing its relevance for downstream functional and systems-level analyses.

In contrast, DECODER-derived profiles showed substantially weaker agreement with the reference, with correlations ranging from r ≈ 0.19 to 0.23 (Figures 8A–D). Although statistically significant, these values indicate a limited capacity to recover astrocyte-specific expression patterns when compared to CDSeq. This discrepancy highlights fundamental differences in how each method models underlying biological signals, with DECODER potentially distributing cell-type–specific signals across latent dimensions, thereby diluting astrocyte-specific transcriptional coherence.

Across all disease stages, CDSeq consistently exhibited higher correlation coefficients with the reference GSE26899 dataset than DECODER, suggesting improved recovery of astrocyte-related transcriptional signals. This pattern remained stable across Control, Incipient, Moderate, and Severe conditions (Figure 8).

From a technical perspective, these findings must be interpreted within the context of non-equivalent data modalities. The validation framework integrates (i) deconvolved bulk transcriptomic profiles and (ii) single-nucleus RNA-seq data obtained from a distinct brain region (entorhinal cortex). This introduces multiple sources of variability, including differences in measurement technology (microarray vs. RNA-seq), tissue-specific transcriptional programs, and the inherent limitations of deconvolution methods, which infer rather than directly measure cell-type expression (Avila Cobos et al., 2020; Wang et al., 2019). Additionally, single-cell data are affected by dropout events and sparsity, whereas deconvolution produces smoothed expression estimates, further contributing to discrepancies in correlation structure.

Within this context, the observed correlation values for CDSeq suggest recovery of biologically coherent astrocyte-associated transcriptional signals despite substantial technical and biological heterogeneity.

Importantly, the incorporation of external snRNA-seq validation in the present study was not intended to replace the unsupervised deconvolution framework, but rather to evaluate whether the inferred latent compartments recover biologically coherent astrocyte-associated signals when compared against independent single-cell–derived references.

To further strengthen the biological interpretation, enrichment analysis of astrocyte-specific transcriptional programs was performed using curated gene sets. CDSeq-derived profiles showed significant enrichment of astrocyte-associated pathways, including glutamate transport, ion homeostasis, and neuroinflammatory response signatures, consistent with established astrocyte functions in the central nervous system.

Interestingly, both methods capture a general trend of increased concordance in intermediate disease stages, particularly in the Moderate condition. This observation aligns with prior studies indicating that astrocytes undergo progressive activation and transcriptional remodeling during Alzheimer’s disease, reaching a peak in intermediate stages before transitioning into more heterogeneous or dysfunctional states in advanced disease (Serrano-Pozo et al., 2024; Escartin et al., 2021).

Taken together, these results demonstrate that CDSeq provides a more accurate and biologically consistent reconstruction of astrocyte transcriptional programs compared to DECODER. While DECODER captures aspects of global transcriptomic variation, its lower correlation and reduced enrichment of astrocyte-specific signals suggest limited specificity in this context, consistent with the stronger astrocyte-specific enrichment observed in Supplementary Figure S3.

## 4 Limitations and future directions

This study has several limitations that should be considered when interpreting the results. First, all analyses are based on a single microarray dataset (GSE28146) with a modest sample size (n = 30), which restricts the generalizability of the findings and precludes robust stratification by additional covariates such as sex, comorbidities, or genetic background. Future studies incorporating larger, multi-cohort datasets will be necessary to assess the reproducibility and population-level robustness of the observed patterns.

Second, the deconvolution algorithms were applied to intensity-based transcriptomic data generated using microarray technology, whereas most contemporary deconvolution frameworks have been optimized for RNA-seq data. As a result, the inferred cell-type-specific expression profiles—and consequently the downstream fluxomic predictions—should be interpreted as biologically informed cross-platform approximations. This technological mismatch introduces inherent limitations related to dynamic range, probe specificity, and signal saturation (Wang et al., 2019), which may affect the precision of inferred biological signals.

Third, flux predictions derived from the astrocyte genome-scale metabolic model were not validated against direct experimental measurements of metabolite concentrations or reaction rates. Therefore, the reported metabolic alterations should be considered as model-based hypotheses rather than quantitative estimates of *in vivo* fluxes.

Furthermore, external validation relied on astrocyte reference profiles derived from the entorhinal cortex, whereas the deconvolution analyses were performed using hippocampal transcriptomic data. Regional differences in astrocyte identity, cellular composition, and disease-associated transcriptional responses may therefore contribute to the attenuation of correlation coefficients observed during external validation.

However, to partially mitigate these limitations, we implemented an external validation framework using independent single-nucleus RNA-seq data from the entorhinal cortex (Serrano-Pozo et al., 2024). This validation demonstrated that CDSeq-derived astrocyte profiles exhibit moderate but biologically meaningful concordance (r ≈ 0.43–0.44) with reference astrocyte expression patterns across disease stages, alongside the preservation of canonical astrocyte markers including GFAP, AQP4, SLC1A2 and SLC1A3, and stronger astrocyte-specific enrichment relative to neuronal, microglial, and oligodendroglial signatures (Supplementary Figure S3). In addition, enrichment analyses confirmed that the inferred signatures capture key astrocyte-related pathways, including glutamate transport and neuroinflammatory responses (Escartin et al., 2021). Importantly, given the cross-platform (microarray vs. RNA-seq), cross-region (hippocampus vs. entorhinal cortex), and methodological (deconvolution vs. single-cell) differences, such correlation values should be interpreted as supportive evidence consistent with the recovery of biologically meaningful astrocyte-associated signals.

Finally, while this study provides a proof-of-concept focused on astrocyte-associated transcriptional and metabolic remodeling in the hippocampus, neurodegeneration is a system-level process involving complex interactions within the neurovascular unit. Extending this framework to include additional cell types (e.g., neurons, microglia, endothelial cells) and brain regions will be essential to capture the full spectrum of metabolic dysregulation. Future work will prioritize the integration of multi-omic and multi-cohort datasets, refinement of metabolic constraints using experimentally derived priors, and the incorporation of spatially resolved transcriptomic data.

## 5 Conclusion

In conclusion, the comparative evaluation of unsupervised transcriptomic deconvolution approaches with genome-scale metabolic modeling represents a powerful strategy to dissect astroglial dysfunction across the continuum from mild cognitive impairment to Alzheimer’s disease. The combined application of CDSeq and DECODER to bulk hippocampal transcriptomes revealed convergent dysregulation in lactate, glutamine, and glutathione pathways, consistent with a progressive collapse of astroglial homeostatic maintenance and antioxidant resilience. These alterations are consistent with a transition from homeostatic to reactive and ultimately dysfunctional astrocyte-associated states, supporting the hypothesis that astrocyte dysfunction contributes to neurodegenerative vulnerability throughout disease progression.

Importantly, the biological validity of the inferred astrocyte signals was supported through external validation using independent single-nucleus RNA-seq data. In this framework, CDSeq-derived profiles showed moderate but robust concordance with reference astrocyte transcriptional signatures (r ≈ 0.43–0.44) across disease stages, alongside preservation of canonical astrocyte markers such as GFAP, AQP4, and SLC1A2, stronger astrocyte-specific enrichment relative to neuronal, microglial, and oligodendroglial signatures, and enrichment of astrocyte-related pathways. Given the cross-platform (microarray vs. RNA-seq), cross-region (hippocampus vs. entorhinal cortex), and methodological (deconvolution vs. single-cell) differences, these results provide convergent evidence that CDSeq captures biologically meaningful astrocyte programs. In contrast, DECODER exhibited lower concordance with snRNA-seq astrocyte reference, suggesting reduced specificity for astrocyte-associated transcriptional signals under these conditions. These findings highlight that technical reconstruction fidelity alone is insufficient to assess deconvolution performance, and that biological validation is essential to determine method reliability. Importantly, these inferred programs should be interpreted as biologically enriched astrocyte-associated compartments derived from heterogeneous tissue deconvolution, rather than as perfectly purified astrocyte transcriptomes.

Within this context, the methodological complementarity between both tools remains relevant. CDSeq leverages probabilistic modeling and Bayesian regularization to generate stable and biologically coherent profiles, whereas DECODER provides increased sensitivity to absolute variation, which may be useful for capturing high-amplitude transcriptional changes. Together, these features support a multi-algorithmic framework in which the robustness of CDSeq and the sensitivity of DECODER can be combined to translate transcriptomic variation into functional metabolic behavior. However, CDSeq consistently demonstrated greater biological specificity and stronger concordance with independent astrocyte reference signatures across the Alzheimer’s disease continuum.

This scalable framework may be extended beyond astrocytes to other glial populations and multicellular representations of the neurovascular unit, enabling cross-cell-type comparisons and system-level analyses of brain metabolism. Future work incorporating larger and more diverse transcriptomic cohorts, multi-omic integration, and improved parameterization of deconvolution algorithms will further enhance the predictive power and biological interpretability of this approach.

Importantly, these results demonstrate that deconvolution methods should be evaluated within a multi-level framework that distinguishes between statistical accuracy and biological validity. This study provides a reproducible strategy for benchmarking reference-free deconvolution approaches and assessing their functional implications through downstream integration with genome-scale metabolic models.

An important limitation of this study is the relatively small and moderately imbalanced sample size of the GSE28146 cohort. Nevertheless, this dataset remains one of the few publicly available human hippocampal transcriptomic resources spanning multiple clinically defined stages across the Alzheimer’s disease continuum, including control, incipient, moderate, and severe conditions. To mitigate potential sensitivity to sampling variability, biological interpretation was supported through multiple complementary analyses, including preservation of canonical astrocyte markers, pathway-level consistency across disease stages, concordance with independent astrocyte single-nucleus RNA-seq references, and downstream metabolic integration. Future studies incorporating larger and more balanced transcriptomic cohorts will be important to further strengthen statistical generalizability and robustness of deconvolution-based metabolic inference in Alzheimer’s disease.

Altogether, this integrative strategy provides a robust and extensible platform for advancing our understanding of astrocyte-centered metabolic dysfunction in neurodegeneration and for identifying potential targets for early intervention and disease-modifying therapies. Expanding this framework to larger cohorts will be essential to further enhance the stability and generalizability of the inferred transcriptomic structures.

## Data Availability

The data presented in this study are publicly available. The transcriptomic dataset analyzed is deposited in the NCBI Gene Expression Omnibus (GEO) repository under accession number GSE28146. The code and additional resources generated during this study are available in the GitHub repository: https://github.com/mangaritar/Deconvolution-Astrocyte.

## Supplementary material

SUPPLEMENTARY TABLE S1 Datasets and reference resources used in this study, including sample sizes, demographic profiles (age), postmortem intervals, associated comorbidities, and specific inclusion criteria for MCI and AD cohorts.

SUPPLEMENTARY TABLE S2 Characterization database of transcriptomic deconvolution tools. This file contains two worksheets. The first sheet catalogs 35 transcriptomic deconvolution tools, detailing their core methodological and operational features, such as input data requirements, tissue compatibility, deconvolution types, mathematical frameworks, programming languages, and computational efficiency. The second worksheet compiles the bibliographic references and original publications for each tool.

SUPPLEMENTARY TABLE S3 Weighting framework and scoring criteria for transcriptomic deconvolution tool evaluation. This matrix outlines the 15 macro-categories and individual features employed to assess tool applicability for hippocampal tissue analysis. Weights range from 1 to 3 based on biological and **technical** relevance, where 3, 2, and 1 represent high, moderate, and low applicability, respectively.

SUPPLEMENTARY TABLE S4 Weighted applicability matrix of transcriptomic deconvolution tools. This dataset presents the final evaluation of the 35 computational tools across 15 macro-categories, with individual scores (1 to 3) were assigned based on the specific criteria established in Supplementary Table S3 to determine tool suitability for hippocampal transcriptomic analysis.

SUPPLEMENTARY TABLE S5 Canonical human astrocyte markers curated from the CellMarker 2.0 database. This file consists of two worksheets. The first sheet cross-references identified astrocyte marker genes across three primary literature sources. The second sheet provides comprehensive metadata for these astrocyte markers, detailing parameters such as species, tissue class/type, gene symbols, and bibliographic support scores.

SUPPLEMENTARY TABLE S6 Quantitative ranking of transcriptomic deconvolution tools based on applicability to the hippocampus. This prioritized list presents the 35 tools systematically evaluated through a combination of scores, highlighting the best-performing and most connected methods in heterogeneous tissue contexts by incorporating topological indices such as connectivity, degree, and betweenness centrality.

SUPPLEMENTARY TABLE S7 Deconvoluted expression matrices of astrocyte marker genes across clinical conditions. This file consists of two worksheets. The first sheet presents the estimated expression profiles of 75 astrocyte marker genes across four progressive clinical stages (Control, Incipient, Moderate, and Severe) derived from CDSeq. The second sheet details the average expression matrix for the same 75 genes obtained through DECODER deconvolution, stratified by the respective clinical cohorts.
