# Supplementary material for: Comparative evaluation of reference-free transcriptomic deconvolution highlights the importance of biological validation in astrocytes across Alzheimer’s disease
Source: Front Bioinform. 2026 Jul 13;6:1858866. doi: 10.3389/fbinf.2026.1858866 (PMC13402868; doi:10.3389/fbinf.2026.1858866)
Supplement: Supplementary file 2 [file Supplementaryfile1.docx]

**
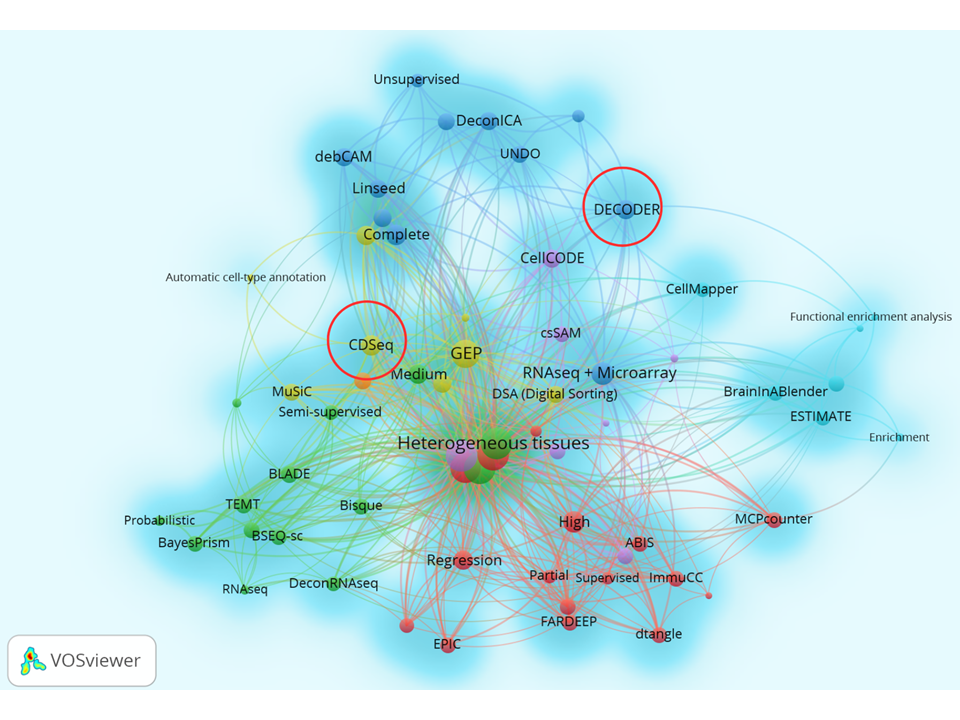
**

**Supplementary Figure S1. Network-based prioritization framework for transcriptomic deconvolution tools.** Bibliometric and methodological relationships among candidate transcriptomic deconvolution approaches were evaluated using a bipartite network topology framework integrating multiple predefined selection criteria. These criteria included: (i) reference dependency (reference-based vs. reference-free modeling), (ii) robustness to heterogeneous tissue composition, (iii) compatibility with bulk transcriptomic datasets, (iv) scalability across sample sizes and computational efficiency, and (v) suitability for downstream integration with genome-scale metabolic models. Each tool was systematically assessed based on curated literature evidence and methodological documentation, and encoded as a feature matrix where edges represent shared methodological properties and benchmarking relationships. Nodes correspond to candidate deconvolution tools, while edge weights reflect the degree of overlap in functional and computational characteristics. Within this framework, DECODER and CDSeq emerged as the most suitable complementary approaches for deconvolution of heterogeneous hippocampal tissue, due to their reference-free modeling strategies, demonstrated robustness under highly variable transcriptomic conditions, and compatibility with downstream metabolic analyses. Their selection reflects a balance between methodological complementarity and biological interpretability in the context of complex neurodegenerative datasets.
