# Supplementary material for: Comparative evaluation of reference-free transcriptomic deconvolution highlights the importance of biological validation in astrocytes across Alzheimer’s disease
Source: Front Bioinform. 2026 Jul 13;6:1858866. doi: 10.3389/fbinf.2026.1858866 (PMC13402868; doi:10.3389/fbinf.2026.1858866)
Supplement: Supplementary file 3 [file Supplementaryfile3.docx]

**
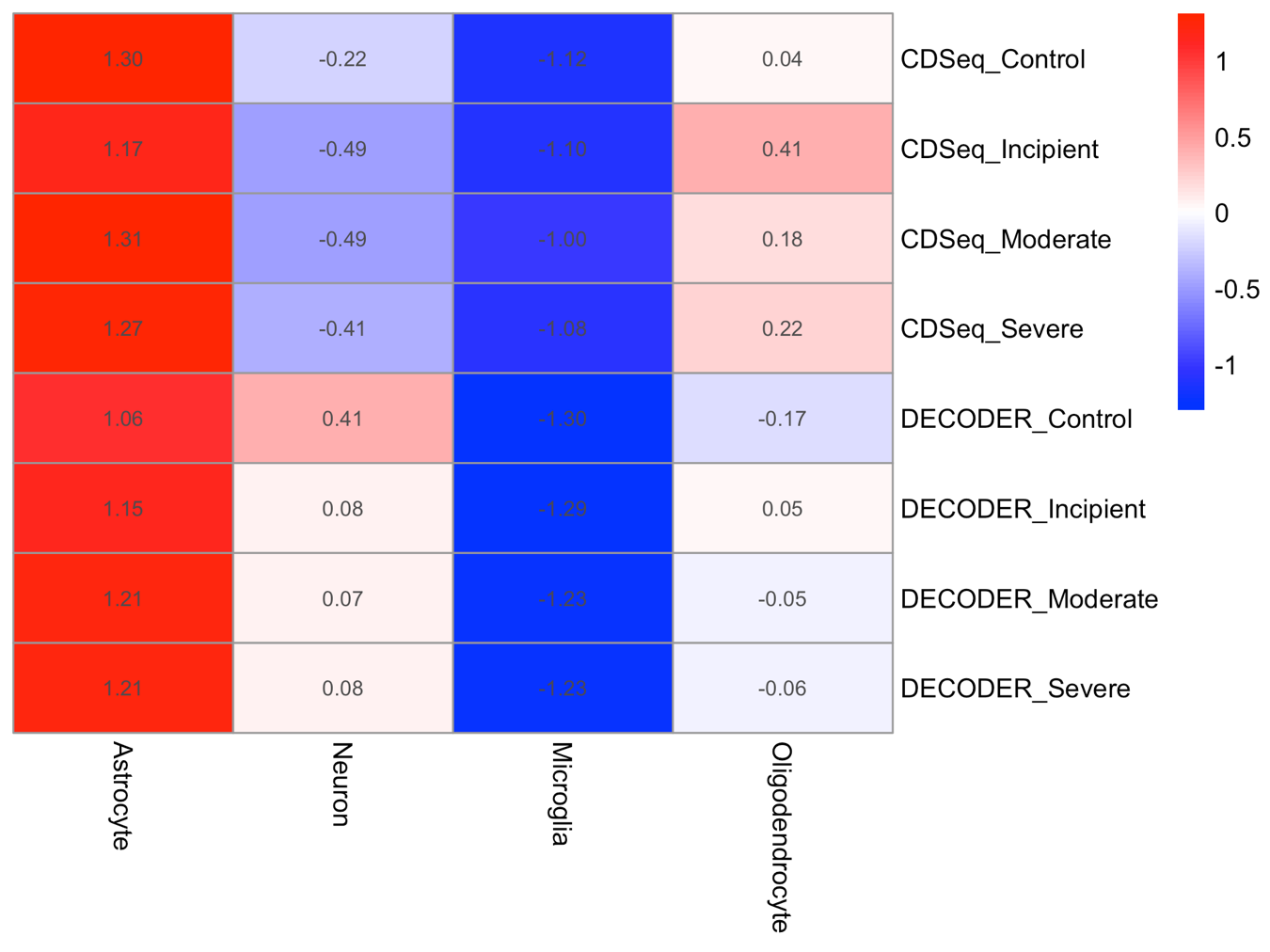
**

**Supplementary Figure S3. Cell-type specificity assessment of deconvolved transcriptomic profiles.** Heatmap showing standardized (z-score) marker gene expression scores for major brain cell types (astrocytes, neurons, microglia, and oligodendrocytes) across disease stages (Control, Incipient, Moderate, Severe) for both CDSeq and DECODER methods. Scores were calculated as the average expression of curated cell-type-specific marker genes. Red indicates relative enrichment, whereas blue indicates relative depletion. CDSeq-derived profiles show strong and consistent enrichment of astrocyte markers with minimal contribution from other cell types, indicating high specificity. In contrast, DECODER-derived profiles exhibit a more heterogeneous pattern with partial contributions from multiple cell types, suggesting reduced specificity and potential signal mixing.
