# Supplementary material for: Comparative evaluation of reference-free transcriptomic deconvolution highlights the importance of biological validation in astrocytes across Alzheimer’s disease
Source: Front Bioinform. 2026 Jul 13;6:1858866. doi: 10.3389/fbinf.2026.1858866 (PMC13402868; doi:10.3389/fbinf.2026.1858866)
Supplement: Supplementary file 5 [file Table3.docx]

**Supplementary Table S3. Weighting framework and scoring criteria for transcriptomic deconvolution tool evaluation.** This matrix outlines the 15 macro-categories and individual features employed to assess tool applicability for hippocampal tissue analysis. Weights range from 1 to 3 based on biological and technical relevance, where 3, 2, and 1 represent high, moderate, and low applicability, respectively.

|  | **Macro-category** | **Characteristics** | **Weight** |
| --- | --- | --- | --- |
| 1 | Input data | RNAseq | 1 |
|  |  | Microarray | 1 |
|  |  | RNAseq + Microarray | 3 |
| 2 | Tissues | Heterogeneous tissues | 3 |
|  |  | Homogeneous tissues | 1 |
| 3 | Cell type capacity | ≥ 5 cell types | 3 |
|  |  | < 5 cell types | 1 |
| 4 | Input requirements | Does not require reference cell-type proportions | 3 |
|  |  | Requires cell-type proportions | 1 |
|  |  | Does not require reference gene expression profiles | 3 |
|  |  | Requires reference gene expression profiles | 1 |
| 5 | Outputs | Cell-type proportions | 3 |
|  |  | Gene expression profiles | 3 |
| 6 | Deconvolution type | Complete | 3 |
|  |  | Partial | 1 |
| 7 | Type | Supervised | 1 |
|  |  | Semi-supervised | 1 |
|  |  | Unsupervised | 3 |
| 8 | Mathematical approach | Regression | 2 |
|  |  | Probabilistic | 3 |
|  |  | Matrix factorisation | 3 |
|  |  | Enrichment | 2 |
|  |  | Convex hull | 2 |
| 9 | Language | R | 3 |
|  |  | Python | 2 |
|  |  | Matlab | 3 |
|  |  | Java | 1 |
|  |  | C++ | 1 |
| 10 | Code availability | Open source | 3 |
|  |  | Restricted | 1 |
| 11 | Application | Stromal cells | 1 |
|  |  | Immune cells | 1 |
|  |  | Blood | 1 |
|  |  | Nervous system | 3 |
|  |  | Tissue mixtures | 1 |
|  |  | Tissue neuronal | 3 |
|  |  | Cancer transcriptome | 1 |
| 12 | Computational efficiency | High | 3 |
|  |  | Medium | 2 |
|  |  | Low | 1 |
| 13 | Additional analytical capabilities | Comparative analysis of orthologous genes across species | 1 |
|  |  | Functional enrichment analysis | 2 |
|  |  | Automatic cell-type annotation | 2 |
|  |  | Rare cell-type annotation | 1 |
|  |  | Applicable to multi-omics data | 1 |
|  |  | Scalable to medium–large cohorts | 1 |
|  |  | Estimation of absolute abundances | 1 |
|  |  | Tumor purity estimation | 1 |
|  |  | Statistical significance estimation | 1 |
|  |  | Flexibility for methylation data handling | 1 |
|  |  | Marker gene identification | 1 |
|  |  | Identification of relationships between cell subtypes | 2 |
|  |  | Prediction of clinical phenotypes and biological processes | 2 |
|  |  | Reconstruction of cellular states and tissue ecosystems | 2 |
|  |  | Visualization of cell-type abundances | 1 |
| 14 | Year (publication) | 2010-2014 | 1 |
|  |  | 2015-2019 | 2 |
|  |  | 2020-2024 | 3 |
| 15 | Number of citations | <50 | 1 |
|  |  | >50 y <100 | 2 |
|  |  | >100 | 3 |
