# Supplementary material for: Comparative evaluation of reference-free transcriptomic deconvolution highlights the importance of biological validation in astrocytes across Alzheimer’s disease
Source: Front Bioinform. 2026 Jul 13;6:1858866. doi: 10.3389/fbinf.2026.1858866 (PMC13402868; doi:10.3389/fbinf.2026.1858866)
Supplement: Supplementary file 7 [file Supplementaryfile2.docx]

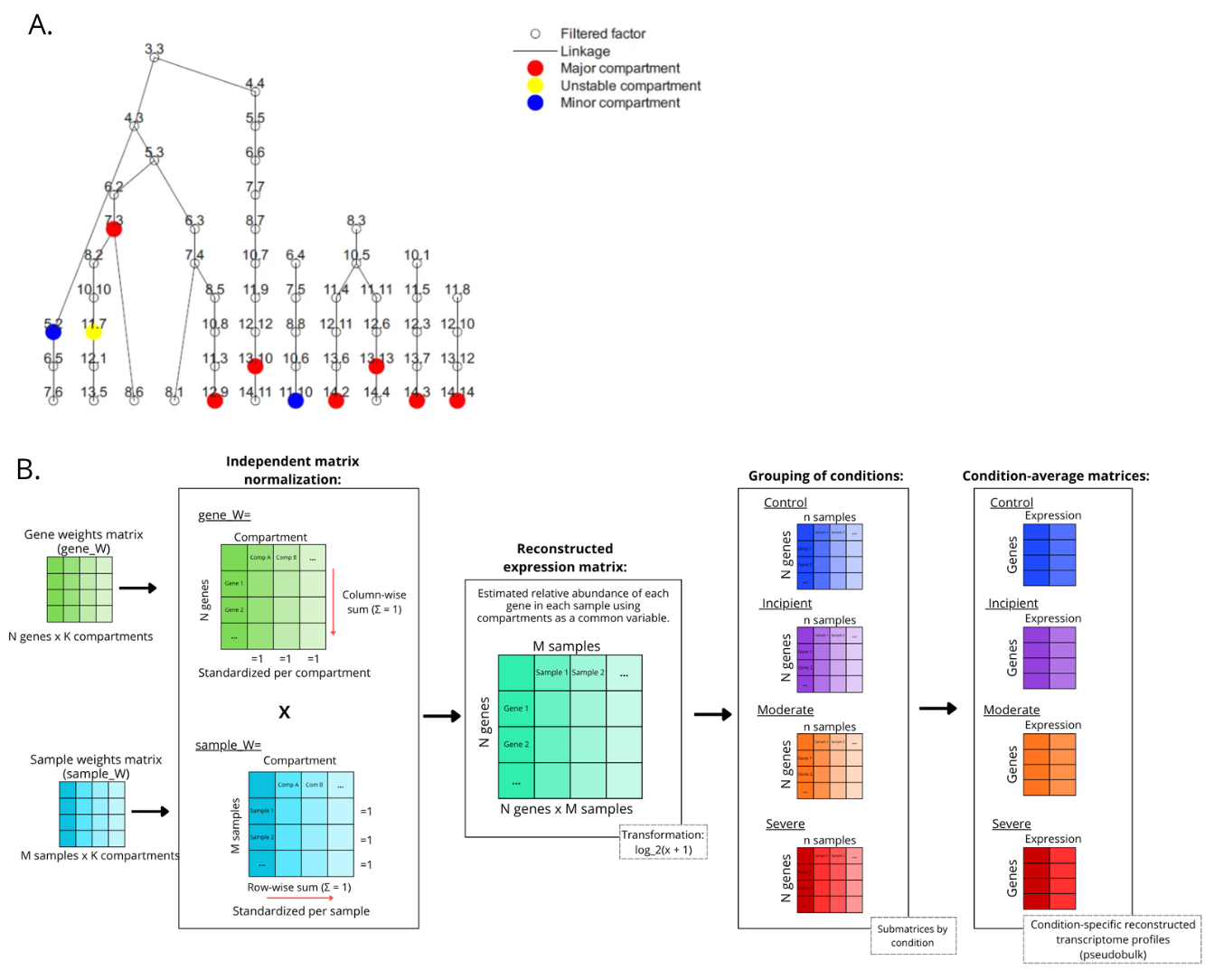


**Supplementary Figure S2. DECODER hierarchical factor tree and downstream methodology for clinical condition stratification.** A. Hierarchical tree of transcriptomic compartments inferred from the unsupervised *de novo* deconvolution of human hippocampal bulk RNA-seq data (N = 30 samples, spanning the CA1–CA3 regions); the deconvolution was executed globally across all unstratified samples. Nodes represent transcriptionally distinct compartments reproducibly identified across multiple clustering runs (K = 2-15) and are color-coded according to their structural stability and biological relevance: red, highly stable and functionally relevant compartments; yellow, compartments with intermediate stability or variability across runs; and blue, poorly represented compartments with low gene density. Each key node is annotated with its primary functional term determined via DECODER-based characterization and Metascape functional enrichment analysis, allowing direct visualization of the biological processes underlying each compartment. Representative annotated modules include: 14.14_MODULE-310 (detoxification and organelle localization); 12.9_STANHILL-HRAS-TRANSFORMATION-UP (protein homeostasis and endoplasmic reticulum stress); 7.3_REACTOME-GABA-A-RECEPTOR-ACTIVATION (GABAergic signaling and synaptic organization); 14.2_GNF2-TPT1 (regulation of translation and cytoskeletal dynamics); 13.13_KANG-DOXORUBICIN-RESISTANCE-DN (proteostasis and cellular stress response); 14.3_REACTOME-GABA-A-RECEPTOR-ACTIVATION (synaptic signaling via GABA receptors); 13.10_MIPS-SMN-POLII-RHA-COMPLEX (proteasome function and translational stress); 11.7_chr9p11 (calcium homeostasis and RTK signaling); 11.10_WEBER-METHYLATED-HCP-IN-SPERM-DN (membrane trafficking and neurodevelopmental processes); 5.2_GNF2-TPT1 (ribosome biogenesis and amino acid metabolism). B. Post-deconvolution downstream workflow for transcriptomic profile reconstruction and pseudobulk generation. Diagram of the analytical process executed to project the deconvolution outputs across different clinical cohorts. Two primary weight matrices derived from DECODER were utilized: the sample weight matrix (sample_w), indicating compartment proportions per sample, and the gene weight matrix (gene_w), defining compartment-specific expression profiles. Each matrix was normalized independently, where gene_w was column-scaled and sample_w was row-scaled (Σ= 1). A global post-deconvolution expression matrix (N genes x M samples) was linearly reconstructed via matrix multiplication (gene_w x sample_w), leveraging the compartments as a common latent variable. Following reconstruction, data variance was stabilized using a log_2_(x + 1) transformation. Finally, sample-level profiles were stratified into their respective clinical cohorts and row-wise averaged, generating four independent pseudobulk profiles tracking disease progression: Control (n = 8), Incipient (n = 7), Moderate (n = 8), and Severe (n = 7).
