# Supplementary material for: Comparative evaluation of reference-free transcriptomic deconvolution highlights the importance of biological validation in astrocytes across Alzheimer’s disease
Source: Front Bioinform. 2026 Jul 13;6:1858866. doi: 10.3389/fbinf.2026.1858866 (PMC13402868; doi:10.3389/fbinf.2026.1858866)
Supplement: Supplementary file 8 [file Table6.docx]

**Supplementary Table S6. Quantitative ranking of transcriptomic deconvolution tools based on applicability to the hippocampus.** This prioritized list presents the 35 tools systematically evaluated through a combination of scores, highlighting the best-performing and most connected methods in heterogeneous tissue contexts by incorporating topological indices such as connectivity, degree, and betweenness centrality.

| **Tool** | **Score** | **Connectivity** | **Centrality** |
| --- | --- | --- | --- |
| **CDSeq** | **51** | **21** | **0.035711** |
| **DECODER** | **48** | **19** | **0.039784** |
| DeconICA | 47 | 18 | 0.027523 |
| TOAST | 45 | 18 | 0.025892 |
| Linseed | 44 | 17 | 0.023211 |
| debCAM | 44 | 18 | 0.044016 |
| Deblender | 43 | 17 | 0.024493 |
| CellCODE | 43 | 17 | 0.028562 |
| CIBERSORTx | 42 | 17 | 0.03505 |
| DeCompress | 40 | 16 | 0.024163 |
| UNDO | 40 | 17 | 0.04661 |
| BayesPrism | 39 | 16 | 0.058991 |
| GEDIT | 38 | 18 | 0.048458 |
| BrainInABlender | 37 | 17 | 0.045533 |
| xCell | 37 | 17 | 0.046065 |
| DSA (Digital Sorting) | 37 | 16 | 0.035244 |
| MuSiC | 37 | 17 | 0.042818 |
| dtangle | 35 | 15 | 0.0215 |
| ABIS | 35 | 16 | 0.02678 |
| BLADE | 35 | 15 | 0.023568 |
| MCPcounter | 35 | 17 | 0.058959 |
| ESTIMATE | 35 | 17 | 0.05852 |
| ImmuCC | 34 | 16 | 0.042966 |
| CellMapper | 34 | 17 | 0.051463 |
| BSEQ-sc | 34 | 16 | 0.03776 |
| FARDEEP | 34 | 15 | 0.027005 |
| CIBERSORT | 34 | 17 | 0.057103 |
| BayesCCE | 33 | 16 | 0.04832 |
| TIMER | 33 | 15 | 0.032857 |
| Bisque | 33 | 16 | 0.050405 |
| csSAM | 33 | 15 | 0.043729 |
| EPIC | 33 | 15 | 0.032709 |
| DeconRNAseq | 32 | 16 | 0.044948 |
| GTM-decon | 32 | 16 | 0.05818 |
| TEMT | 32 | 15 | 0.033004 |
